# Quantifying forest disturbance regimes within caribou (*Rangifer tarandus*) range in British Columbia

**DOI:** 10.1038/s41598-024-56943-0

**Published:** 2024-03-19

**Authors:** James C. Maltman, Nicholas C. Coops, Gregory J. M. Rickbeil, Txomin Hermosilla, A. Cole Burton

**Affiliations:** 1 Department of Forest Resources Management, Faculty of Forestry, University of British Columbia, Vancouver, BC Canada; 2 Ecofish Research, Suite 303-2012 Washington Street, Rossland, BC V0G 1Y0 Canada; 3 Canadian Forest Service (Pacific Forestry Centre), Natural Resources Canada, 506 West Burnside Road, Victoria, BC V8Z 1M5 Canada

**Keywords:** Forest ecology, Macroecology

## Abstract

Habitat disturbance is a major driver of the decline of woodland caribou (*Rangifer tarandus caribou*) in Canada. Different disturbance agents and regimes negatively impact caribou populations to different degrees. It is therefore critical that land managers and scientists studying caribou have a detailed understanding of the disturbance regimes affecting caribou habitat. In this work we use recent advances in satellite-based disturbance detection to quantify polygonal forest disturbance regimes affecting caribou ecotypes and herds in British Columbia (BC) from 1985 to 2019. Additionally, we utilize this data to investigate harvesting rates since the implementation of the Species at Risk Act (SARA) and publication of recovery strategies for caribou in BC. Southern Mountain caribou herds are the most threatened yet experienced the highest rates of disturbance, with 22.75% of forested habitat within their ranges disturbed during the study period. Over the study period, we found that in total, 16.4% of forested area was disturbed across all caribou herd ranges. Our findings indicate that caribou in BC face high, and in many cases increasing, levels of habitat disturbance. Our results provide a detailed understanding of the polygonal disturbance regimes affecting caribou in BC at the herd scale, and highlight the need for effective implementation of policies aimed at preserving caribou habitat.

## Introduction

Disturbance is a critically important process in forest ecosystems, with major impacts on forest species^1,2^. Human influence, both direct and indirect, is altering the frequency, duration, size, and number of disturbance events in forested environments^3,4^. Habitat loss as a result of disturbance has been linked to reductions in species abundance, as well as slowed recovery of endangered species^5–9^. Proper implementation of management strategies for species at risk which maintain viable populations in the face of dynamic and increasing cumulative disturbance, require a comprehensive understanding of the current disturbance regimes affecting these species, and how these regimes are changing.

Understanding and managing disturbances is particularly important for the conservation of woodland caribou (*Rangifer tarandus caribou*), a culturally and ecologically important threatened subspecies in Canada which are highly impacted by disturbance^10–12^. Despite significant efforts to recover populations, including multiple listings on Schedule 1 of the Species at Risk Act (SARA) in 2004, and the publication of recovery strategies in 2012 and 2014, woodland caribou populations are still declining across their range in British Columbia (BC), as well as Canada wide^13–18^. While drivers of caribou population decline are complex, major factors include habitat alteration and increased predation due to disturbance-mediated apparent competition^17,19,20^. In the recovery strategy for the Boreal ecotype of woodland caribou, the Canadian government identified a requirement for at least 65% of habitat in a herd range to remain undisturbed in order to have a 60% probability of the herd to be self-sustaining, with this threshold often applied to other caribou ecotypes in BC^15,17^.

Many studies have investigated the impact of forest disturbance on caribou habitat and population dynamics, finding that both natural and anthropogenic disturbances are negatively correlated with caribou habitat use and population stability^10–12,21,22^. Some studies have found fire to be significantly less detrimental to caribou populations than anthropogenic disturbance^12,21^. Caribou disturbance studies typically use forest industry and government disturbance records to quantify the occurrence and intensity of disturbance^10,21,22^. While these disturbance records give a general understanding of disturbance dynamics, they are typically compiled from a number of agencies using differing standards, and can be impacted by missing data in remote areas, bias towards larger disturbances, and lack of information regarding certain disturbance agents^23–25^. Non-stand replacing disturbances including biotic (e.g. insect infestation) and abiotic (e.g. drought) disturbances, are only coarsely mapped across BC. Forest health surveys are typically focused on a limited number of observable biotic disturbances, identified from aircraft and manually annotated on maps^26^. Disturbances not clearly observable from the air are often missed. Similarly, there is no mapping of abiotic non-stand replacing disturbances such as drought or windthrow^26^. While these surveys provide useful overview information at a provincial scale and inform on general trends, the assessments are subjective and relatively coarse in spatial detail, hampering analysis of fine scale trends^26,27^.

The number of different data sources used to conventionally identify disturbance also makes direct comparison of disturbance levels between areas and disturbance agents difficult. Information on harvesting levels in BC, for example, is derived by combining information from the National Forest Inventory (NFI), Vegetation Resources Inventory (VRI), and Landsat-derived change detection from 1991 onwards, each with their own methodology for identifying disturbance^28^. The same is true for fire records, with fire perimeters derived from a combination of aerial surveys, GPS digitization, and point buffers for historical fires, and aerial photography and satellite data in more recent years^29^. These methodological differences can have large impacts on calculation of total area disturbed, with historical methods of fire perimeter delineation found to overestimate total fire area by 11% across Canada^29^. These differences make it difficult to directly compare disturbance levels between different disturbance agents, as well as between different areas where different methodologies for disturbance identification may have been used. A methodologically consistent, spatially explicit method of disturbance detection is therefore desirable for a comprehensive, wall-to-wall analysis of disturbance regimes affecting wide-ranging species such as woodland caribou.

Traditional estimates of forest disturbance are typically polygon-based, aggregating disturbances at a relatively coarse spatial scale; they therefore do not inform on the patch dynamics of disturbance, such as remnant unburned areas of a fire^27,30^. Patch dynamics are a useful indicator of habitat fragmentation, which has been shown to be potentially detrimental to caribou habitat use^31,32^. Additionally, patch dynamics are directly tied to the calculation of undisturbed critical habitat under the recovery strategy for Boreal caribou, with anthropogenic disturbances being buffered by 500 m to calculate total disturbed area under the strategy^17^. Thus, for example, a forest harvesting regime comprised of many, small logged areas would constitute a larger total area disturbed under the strategy than a landscape comprised of fewer, larger logged areas.

With the advent of free- and open-access to the Landsat archive, marked advances in automatic change detection have occurred which offer advantages compared to these existing disturbance datasets^33–37^. Many studies have utilized these new datasets to quantify forest disturbance regimes, determining disturbance frequency and trends^26,30,34,36,38,39^. Disturbance information derived from satellite imagery provides the opportunity to characterize disturbance regimes through time with a consistent and spatially explicit methodology and associated validation, providing wall-to wall coverage of the study area, using the same methodology to identify and describe every disturbance agent^40,41^. These disturbance products are well-suited for characterizing polygonal disturbance regimes from an ecological perspective, allowing for comprehensive coverage of remote areas, information on the spatial distributions and characteristics of disturbance, and analysis of changes in disturbance patterns through time^40^.

In a previous study investigating disturbance in caribou range, Nagy-Reis et al.^42^, used a satellite-derived forest change data product to estimate change in treed area in caribou habitat in Alberta and BC, with the BC government using these results, among many other data sources to track disturbance rates^43^. However, more study regarding satellite-derived disturbance monitoring is needed. While Nagy-Reis et al.^42^, quantified stand-replacing disturbance from 2000 to 2018, disturbances were only attributed to causal agents within a relatively short time horizon (2000–2015), and patch dynamics, i.e. the average size and number, of these disturbances, were not evaluated. Non-stand replacing disturbance (NSR), such as insect attack was also not investigated. A longer time horizon, deeper investigation into the patch dynamics of disturbance, and analysis of non-stand replacing disturbance will augment targeted policymaking and management to arrest and reverse the decline of caribou in BC.

In this study we utilize openly available wall-to-wall forest disturbance data over BC to characterize and investigate changes in the disturbance regimes affecting caribou at the herd level from 1985 to 2019. Using forested area as a proxy for caribou habitat^42,44,45^, we examine forested area disturbed and disturbance event patch dynamics within caribou herd polygons at an annual time step for stand-replacing (i.e., fire, harvesting) and non-stand replacing disturbances. We then examine how these variables have changed over the study period. Analysis is conducted at a 30 m scale with a 0.5 ha minimum mapping unit, recognizing that at this spatial scale, while polygonal disturbances are accurately delineated, fine linear features such as roads and seismic lines associated with oil and gas exploration will often be missed.

Due to these constraints, we do not consider linear features in this analysis, and only evaluate polygonal disturbance, highlighting that linear disturbances have significant negative impacts on woodland caribou^46^. The omission of these linear features will result in more missed disturbance in northern herds with disturbance regimes more dominated by oil and gas exploration than for southern herds more impacted by forestry and fire^12,42,47^.

Availing on the long period of data availability afforded by the Landsat record, we investigate disturbance rates in caribou ranges during the lag time between SARA implementation and the publication of recovery strategies/identification of Critical Habitat, as well as since the publication of recovery strategies and Critical Habitat designation. The long time-frame and high accuracy of the disturbance product utilized in this study allow for a comprehensive examination of polygonal disturbance regimes within caribou range in BC, with the goal of informing pressing management decisions about where habitat protection and restoration efforts are most urgently needed.

## Study area

Due to variable physiography and climate, the ecology of BC is highly diverse. On the western side of the province lies the Pacific Ocean and Coast Mountains, and on the east, the Rocky Mountains, with a large central interior plateau in the center. Environments range from wet, highly productive forests on the coast, to arid steppes in the southern interior, to areas of muskeg and black spruce in cold northern mountains^48^. Woodland caribou utilize a number of these environments, including subalpine forests, mature low elevation forests, peatlands, muskegs and alpine ridges^15,17^.

**Figure 1:**
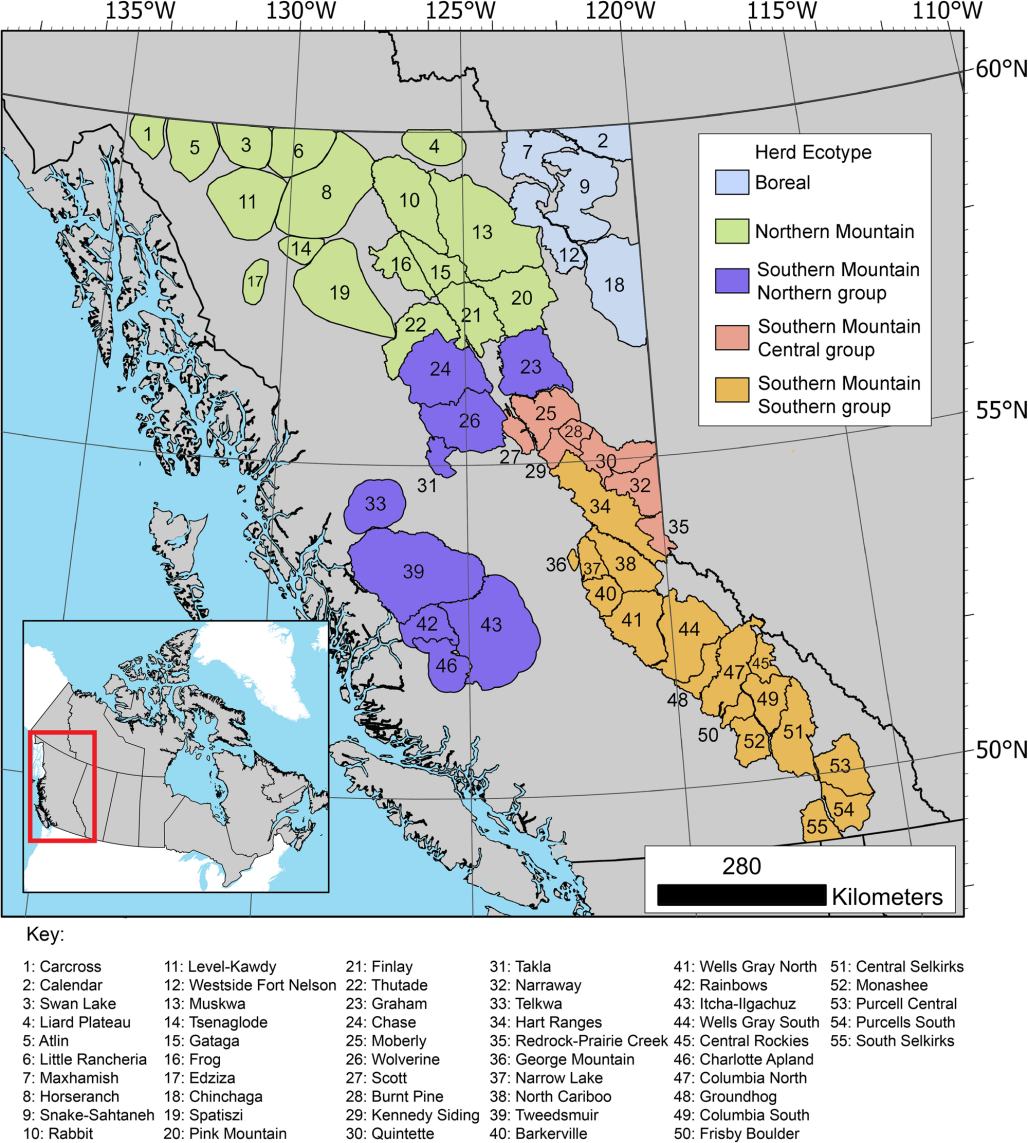
Map of Caribou herds in British Columbia, labelled by ecotype. Maps were generated in ArcGIS Pro (version 3.2.0)^77^.

Caribou range in BC is divided into 55 herds (Fig. 1). Herd boundaries span from the southern to northern border of the province, covering a sum area of 4 Mha^49^. While habitat use varies, all caribou in the province require large, undisturbed areas of mature and old forest to maintain a stable population^15,17^.

BC’s forests experience a large number of disturbances, both natural, and anthropogenic. Forest harvesting is a frequent activity, especially in the south and center of the province, with forestry being a major part of the province’s economy^50^. A decline in market conditions around 2006 led to a decrease in timber harvesting throughout the 2000’s. Recently, harvesting in some areas has increased as a result of salvage logging in response to the mountain pine beetle outbreak affecting much of the province^51^.

Fire is a frequent disturbance affecting many of BC’s forests. Historically dominated by mixed-severity fires, many of BC’s interior forests are now experiencing significantly increased levels of fire severity as a result of climate change and nearly a century of fire suppression^52^. Back-to-back record-breaking fire seasons occurred in 2017 and 2018, with 1.2 Mha of forest burned in 2017, followed by 1.35 Mha burned in 2018^53^.

A severe outbreak of endemic mountain pine beetle affected BC from the 1990’s though the 2000’s, majorly impacting ecosystems throughout the interior of the province. Mountain pine beetle attacks lodgepole pine stands, which dominate much of the interior of BC^54^. This beetle outbreak has had knock-on effects for both harvesting and fire, with salvage logging to recover impacted timber significantly increasing in recent years^55,56^. Additionally, in areas where harvesting is impractical, increased fuel loading in certain stages of beetle attack has lead to increased fire risk^57^.

## Data and methods

### Data

#### Landsat-derived datasets

Forest disturbance layers from 1985 to 2019, derived from Hermosilla et al.^58^ were used as the main driver for characterizing disturbance regimes. They were created from annual Landsat surface reflectance composite, generated using the Composite2Change (C2C) approach developed by Hermosilla et al.^58^. C2C utilizes the freely accessible Landsat archive to create annual, gap-free, surface-reflectance image composites with a 30-m spatial resolution. Pixel scoring functions are applied to all atmospherically corrected Landsat images acquired within 30 days of August 1st (for centrality to the growing season) to produce best available pixel surface reflectance image composites. These scoring functions facilitate selection of the best-available pixel observations annually, based on factors such as sensor type, day of acquisition, distance to cloud or cloud shadow, and atmospheric opacity^59^. Due to sparse data availability and persistent clouds in certain areas, gaps are present in these annual Landsat surface reflectance composites. Gaps are filled with synthetic proxy values of surface reflectance assigned through a spectral trend analysis of the Normalized Burn Ratio (NBR) index^60^.

Discrete change events are detected using a bottom-up breakpoint detection algorithm over an NBR time series for each pixel from the annual Landsat surface reflectance composites. This step is followed by a spatial analysis to avoid inconsistencies which may arise from the pixel-based compositing process, as well as the application of a 0.5-ha minimum-map-unit filter, to remove disturbance events smaller than this filter^58^. Detected changes are then labelled by agent using a Random Forests modeling approach that incorporates spectral, temporal, and object-level (i.e., disturbance patch) information^34^.

Three causes of forest disturbance (disturbance agents) were considered in this research: fire, harvest, and non-stand replacing disturbance (NSR), with harvest referring to forest harvesting such as logging, and NSR referring to changes in vegetation over time that do not lead to change in land-cover class, such as insect infestation, drought stress, and disease. The overall accuracy of C2C change detection reported by Hermosilla et al.^58^ was 90%. Temporally, 89% of changes were detected in the correct year and 98% were within ± 1 year. Changes were attributed with an overall accuracy of 92%. Stand-replacing disturbances (i.e., fire, harvesting) were detected more reliably (detection rate > 95%) than NSR disturbances (83%)^58^.

Forested area was defined using a 30-m spatial-resolution forest mask layer, derived with the methodology outlined in Wulder et al.^61^ for the year 2019, following the UN Food and Agriculture Organization (FAO) definition of forest^62^. While treed area is the area currently covered by trees, forest area was defined by applying temporal and spatial rules to meet the FAO definition of forest over annual land cover^33^ and disturbance layers. All treed areas in 2019 outside of Agriculture and Agri-Food Canada agricultural mask were considered forested. In addition, all areas that were treed prior to a stand-replacing disturbance within the data record (1984–2019) were considered forested, as they are expected to recover and become treed again^61^.

#### Caribou herd polygons

Publicly available herd boundary polygons from the BC Caribou Recovery Program^49^ were utilized to define the locations of caribou herds in BC. Herd was defined as synonymous with subpopulation. Herd boundaries were delineated in the product based on the area required for a herd to be self-sustaining, and were drawn from current science and expert knowledge^49^. The layer contains all 55 herds located in BC, including five currently extirpated herds located mainly in the south of BC. Though some herds share portions of their range, herd boundaries are drawn so as not to overlap. For some herds, full herd ranges extend outside of BC, but are not included in these polygons. Herds are divided into three ecotypes within British Columbia under the Species at Risk act, based on ecological and evolutionary differences: Boreal, Northern Mountain, and Southern Mountain, with Southern Mountain being further divided into Southern, Central, and Northern groups^15,17,63^.

#### Caribou population data

Caribou population data was utilized to estimate herd declines. Recent population estimates were drawn from publicly available 2021 population estimates from the BC government (BC Caribou Recovery Program, 2021), while previous population estimates were drawn from reported numbers in the Southern Mountain caribou recovery strategy and Northern Mountain caribou management plan. The Boreal caribou recovery strategy did not report population numbers, and thus was not included^15,17,63^.

### Methods

Detected disturbance events derived from the Landsat disturbance dataset were defined as spatially contiguous collections of pixels with the same disturbance agent and year of disturbance. Three disturbance agents were considered: fire, harvest, and NSR. To quantify disturbance regimes, disturbance characteristics were assessed at differing spatial scales by annually stratifying by ecotype/group, and then by herd. This approach provides information on both disturbance regimes affecting individual herds, but also broader trends in disturbance across caribou range. Disturbance regimes were characterized using several metrics. To characterize total disturbance levels affecting herds, annual area disturbed by disturbance agent was calculated. Disturbed pixels in forested areas were annually summed by herd. Multiple disturbances over time in the same pixel were considered, in order to account for the possibility of regrowth and re-disturbance, as well as NSR leading into other disturbances such as fire or salvage logging. Percent of forested area disturbed was calculated by dividing forested area disturbed by the total forested area by herd. Disturbance cycle, a metric commonly used to calculate the number of years it would take for an area equivalent to the study area to be disturbed under the current disturbance regime^64^, was calculated following Bond and Keeley^65^ as the reciprocal of the average percent area annually disturbed. These metrics inform on levels of disturbance by herd and ecotype through time, and which disturbance agents have had the greatest impact on habitat. The level of habitat fragmentation caused by differing disturbances in each herd range was assessed by summarizing the annual distribution in size and number of disturbance events by agent within herd boundaries.

To evaluate trends in disturbance levels through time, the rate of change of area disturbed was calculated over differing epochs and over all disturbance agents: the latest decade of the study period (2010–2019), the decade prior (2000–2009), and the entire study period (1985–2019). Rate of change was calculated using non-parametric tests to account for non-normality of data, the Theil-Sen trend estimator was used to calculate rate of change, and significance was calculated using the Mann–Kendall trend test^66–69^. Percent rate of change of disturbed area was calculated as the rate of change divided by average annual area disturbed. Rate of change was also assessed for average disturbance event size and number of disturbance events. Due to the relative infrequency of disturbance events for some herds, rate of change for these was only calculated by caribou ecotype. Results for all herds are available in supplementary Tables 1, 2, 3.

We investigated disturbance rates before and after two major milestones for caribou conservation in Canada; the full implementation of SARA in 2004, and the publication of recovery strategies and management plan for caribou in BC in 2012 and 2014. SARA was fully implemented in 2004, and listed both Boreal and Southern Mountain caribou as threatened. The recovery strategy for Boreal caribou, and management plan for Northern Mountain caribou were published in 2012, with the recovery strategy for Southern Mountain caribou published in 2014. The goal of these documents was to prescribe measures for the preservation and recovery of caribou population levels^15,16,63^. Consequently, Critical Habitat was identified for both ecotypes in these recovery strategies (though only partially for Southern Mountain Caribou)^14^. Northern Mountain caribou were listed as of Special Concern under SARA in 2005, and thus did not receive any designated Critical Habitat^63^. We evaluated harvesting levels specifically, as they are an anthropogenic disturbance, and thus most likely to be immediately and directly impacted by policies aimed at mitigating disturbances.

While critical habitat was not identified until later, and only partially identified for Southern Mountain caribou, the implementation SARA in 2002 provided opportunities for habitat protection, such as section 80 orders, which allow the federal government to directly protect habitat on non-federal lands. Although never used for caribou^70^, the potential implementation of this section, as well as knowledge of the upcoming designation of critical habitat, may have motivated a reduction of harvesting activity, thus we examined whether harvesting levels for any caribou ecotype declined after SARA implementation.

Annual area harvested was grouped into three epochs based on year of observation: pre-SARA (1985–2004), post-SARA (2005–2012/2014, and post-recovery strategy implementation (2013/2015–2019). The pre-SARA epoch represents a period before which national-level policy regarding species at risk was implemented. The post-SARA epoch represents a transitional period where SARA was extant, but some critical documents and data such as recovery strategies and critical habitat designations were not yet complete, while the post-recovery strategy period represents a period after which critical habitat had been identified, and some protection measures regarding critical habitat began to be implemented. The long timeframe of the pre-SARA epoch affords a baseline of harvesting activity before the implementation of any national policy. A Generalized Least Squares approach was used to determine if there were statistically significant (P < 0.05) differences in harvesting levels between any time period. Temporal autocorrelation between observations was accounted for by including an AR(1) autocorrelation term for year of observation.

Analysis was performed in R (version 4.3.0)^71^ using the Tidyverse (version 2.0.0)^72^, Robslopes (version 1.1.3)^73^, and Kendall (version 2.2.1)^74^ packages for statistical analysis, and the Terra (version 1.7)^75^ package for spatial analysis. Figures were generated in R (version 4.3.0)^71^ using the ggplot2 (version 4.3.2)^76^ package. Maps were generated in ArcGIS Pro (version 3.2.0)^77^.

## Results

Over the study period (1985–2019), a total of 16.4% of forested area was disturbed over all caribou herd ranges. For Southern Mountain caribou, 22.8% (3,641,500 ha) of forested area was disturbed, 9.0% (817,999 ha) of forested area was disturbed for Northern Mountain caribou, and 6.6% (238,363 ha) of forested area was disturbed for Boreal caribou. Southern Mountain Northern group caribou were affected by the most disturbance of any Southern Mountain group, at 29.48% of forested area (383,739 ha). Boreal, Northern Mountain, and Southern Mountain Northern group caribou had disturbance regimes primarily driven by fire, while Southern Mountain Central, and Southern Mountain Southern group caribou had disturbance regimes dominated by harvesting (Table 1).

**Table 1:** Mean annual area disturbed by disturbance agent, mean annual number of disturbance events, mean size of disturbance events, and Theil-Sen Test results for the three caribou ecotypes in British Columbia.

Ecotype	Mean annual area [ha/year]	Percent annual area [%/year]	Disturbance cycle [years]	Rate of change [ha/year]			Mean annual number of events	Rate of change 1985–2019 [count/year]	Mean event size [ha]	Rate of change 1985–2019 [ha/year]
				2010–2019	2000–2009	1985–2019				
Fire										
Boreal	4400.7	0.12%	819			36.99 (0.82%)	51	1.5 (2.93%)	205.6	3.6 (1.73%)
Northern Mountain	12,341.6	0.14%	733			133.92 (1.06%)	102		582.0	
Southern Mountain Northern	23,502.5	0.33%	304			305.06 (1.26%)	26		103.5	
Southern Mountain Central	2,027.9	0.09%	1,072				174	3.3 (1.89%)	359.0	
Southern Mountain Southern	3474.3	0.05%	1914				77		255.2	
Harvesting										
Boreal	1327.0	0.04%	2715			− 53.98 (− 4.1%)	73	− 1.8 (− 2.39%)	58.1	− 1.4 (− 2.42%)
Northern Mountain	1135.7	0.01%	7962		− 222.68 (− 19.74%)	− 43.47 (− 3.85%)	87		79.8	− 1.4 (− 1.82%)
Southern Mountain Northern	17,901.2	0.25%	399	882.2 (4.86%)	− 1617.66 (− 8.91%)		288	2.3 (0.81%)	131.0	
Southern Mountain Central	5,835.7	0.27%	372	317.91 (5.44%)			1,075		152.1	
Southern Mountain Southern	19,658.1	0.30%	338	1286.57 (6.67%)	− 897.34 (− 4.65%)	− 354.45 (− 1.84%)	1353	− 16.5 (− 1.22%)	251.5	
Non-stand replacing										
Boreal	1082.7	0.03%	3328				442		10.9	
Northern	9894.0	0.11%	914				3069		46.0	
Southern Mountain Northern	18,788.3	0.26%	380				828		19.8	
Southern Mountain Central	3100.3	0.14%	701				4478		25.0	
Southern Mountain Southern	9754.5	0.15%	682			− 326.1 (− 3.51%)	2760.3	− 109.1 (− 3.95%)	44.4	

Rates of change are only presented for trends with p < 0.05.

Boreal and Northern Mountain ecotype caribou experienced increasing rates of annual area disturbed by fire from 1985 to 2019 increasing by 36.99 ha annually for Boreal caribou, and 133.9 ha annually for Northern caribou (Table 1). All three ecotypes experienced small to moderate decreases in annual area harvested from 1985 to 2019, with the Southern Mountain ecotype seeing a decrease of 2613 ha/year from 2000 to 2009, followed by a 3093 ha/year increase from 2010 to 2019. Northern Mountain ecotype caribou experienced a large 19.7% decrease in area harvested from 2000 to 2009 (Table 1).

Disturbance agents varied by group within the Southern Mountain ecotype. All three groups (Southern, Central, and Northern) had high levels of harvesting, between 0.25% and 0.3% of area annually. Moderate variation between groups was present in levels of NSR, at 0.15% of area annually for the Southern group, 0.14% for the Central group, and 0.26% for the Northern group. Annual area disturbed by fire varied widely, with 0.33% of area being annually disturbed by fire for the Northern group, but only 0.05% of area annually disturbed by fire for the Southern group. An increase in harvesting levels in the last decade was found for all three groups, with the Southern group seeing the largest at 6.67% (Table 1).

**Figure 2:**
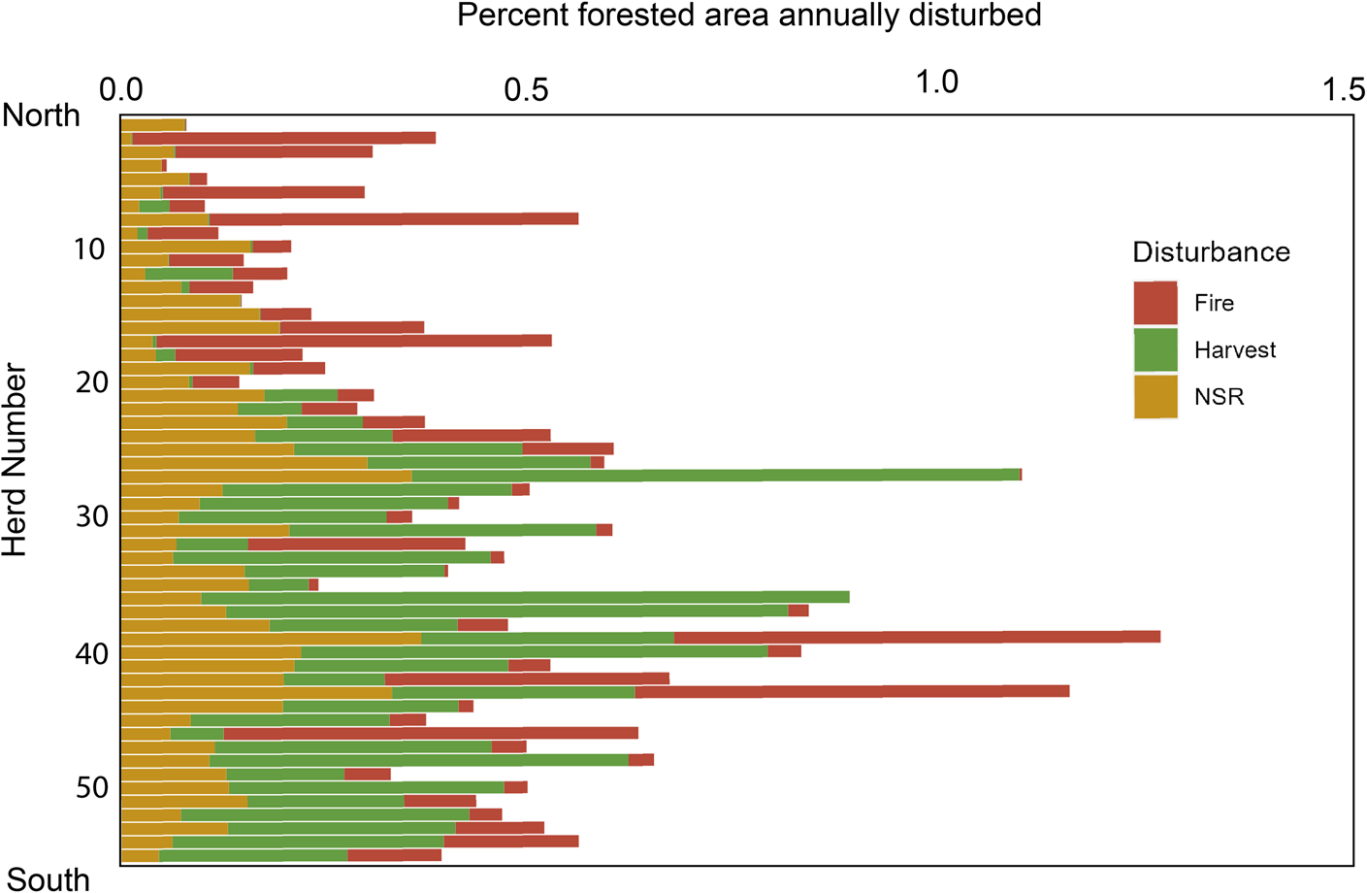
Average percent of forested area annually disturbed by agent for all caribou herds in British Columbia, ordered from North to South. See Fig. 1 for herd names.

**Figure 3:**
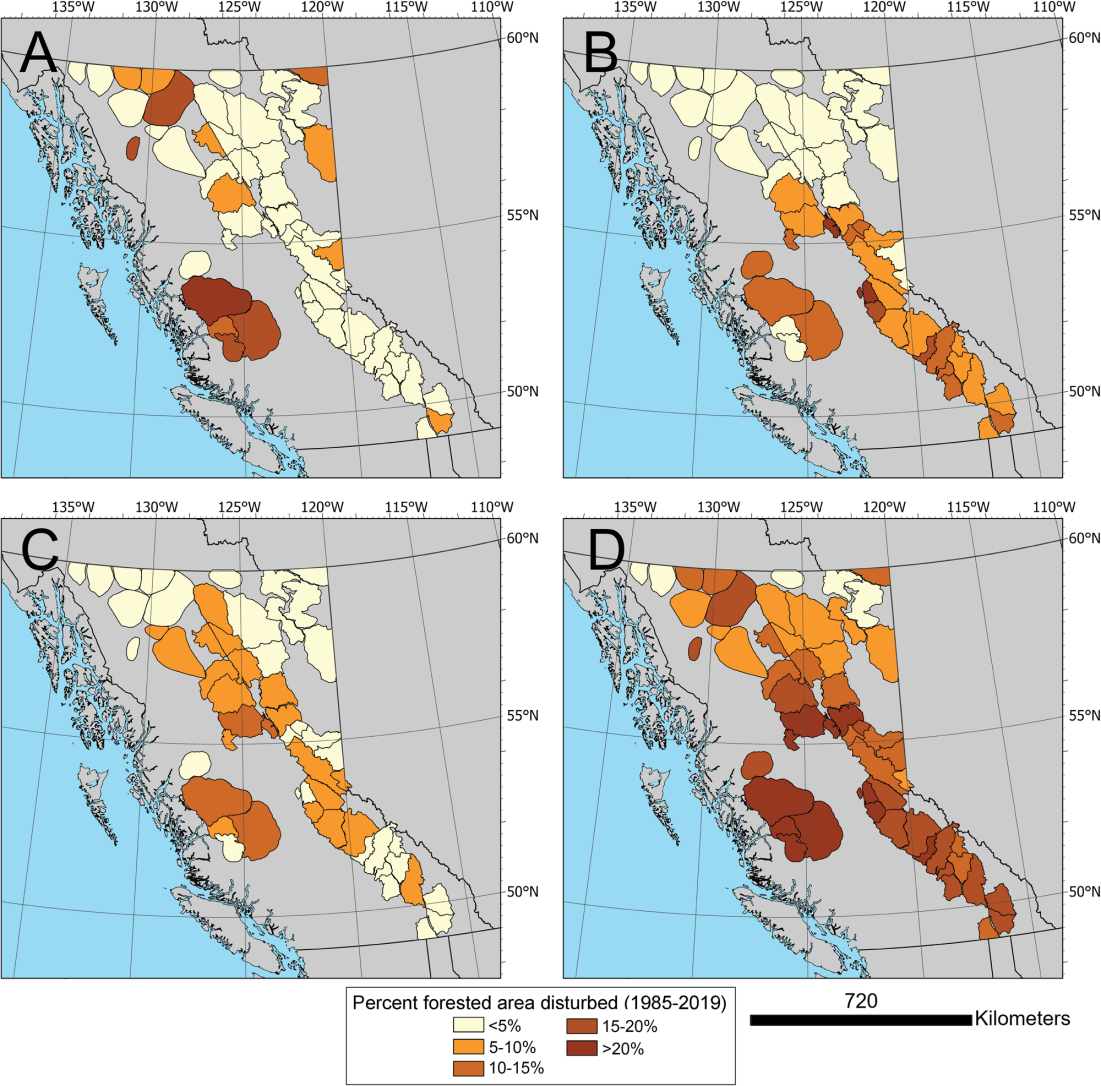
Percent forested area disturbed from 1985 to 2019 by (**A**) Fire, (**B**) Harvest, (**C**) Non-stand replacing disturbance and (**D**): Total. Maps were generated in ArcGIS Pro (version 3.2.0)^77^.

Herds in the north of the province tended to have disturbance regimes driven by fire and non-stand replacing disturbance, with less overall disturbance, while herds to the south tended to have harvest- and fire-driven disturbance regimes with disturbances occurring more frequently (Figs. 2, 3). Over 35% of forested area was disturbed over the study period for three herds: the Tweedsmuir (44.5%), Itcha Ilgatchuz (40.6%), and Scott (38.6%) herds. For three more herds, at least 25% of forested area was disturbed over the study period: the George Mountain (31.2%), Narrow Lake (29.4%), and Barkerville (29.1%) herds.

**Figure 4:**
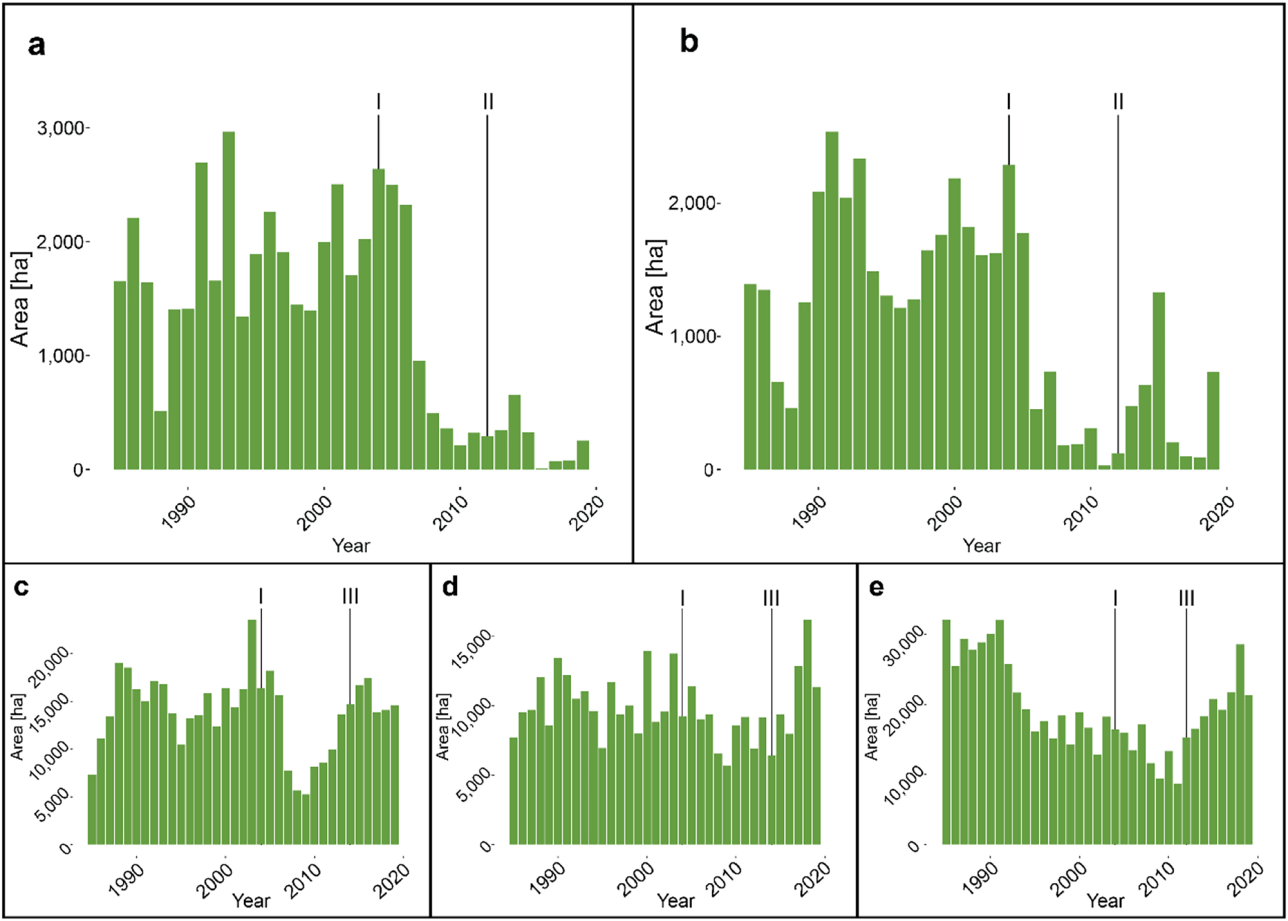
Annual area harvested for (**a**) Boreal, (**b**) Northern Mountain, (**c**) Southern Mountain Northern, (**d**) Southern Mountain Central, and (**e**) Southern Mountain Southern caribou. Lines representing: I: Full implementation of the Species at Risk Act in 2004, II: 2012 publishing of management plan for Northern Mountain caribou and recovery strategy for Boreal caribou, III: 2014 publishing of recovery strategy for Southern Mountain caribou.

For all groups of Southern Mountain caribou, no statistically significant difference was found between harvesting levels in the pre-SARA epoch and harvesting levels in the post-SARA, or post-recovery strategy epoch. For both Boreal and Northern Mountain caribou, we found a statistically significantly lower level of area annually harvested between the pre-SARA epoch and the post-SARA as well as the post-recovery strategy epoch. For Boreal caribou, average area annually harvested in the post-SARA epoch was 46.8% lower than it was from the pre-SARA epoch. For Northern mountain caribou, average area annually harvested in the post-SARA epoch was 62.4% lower than it was in the pre-SARA epoch. Harvesting levels did not immediately decline after the implementation of SARA but rather fell a few years later (Fig. 4). We found no statistically significant difference in harvesting levels between the post-SARA epoch and the post-recovery strategy epoch.

**Figure 5:**
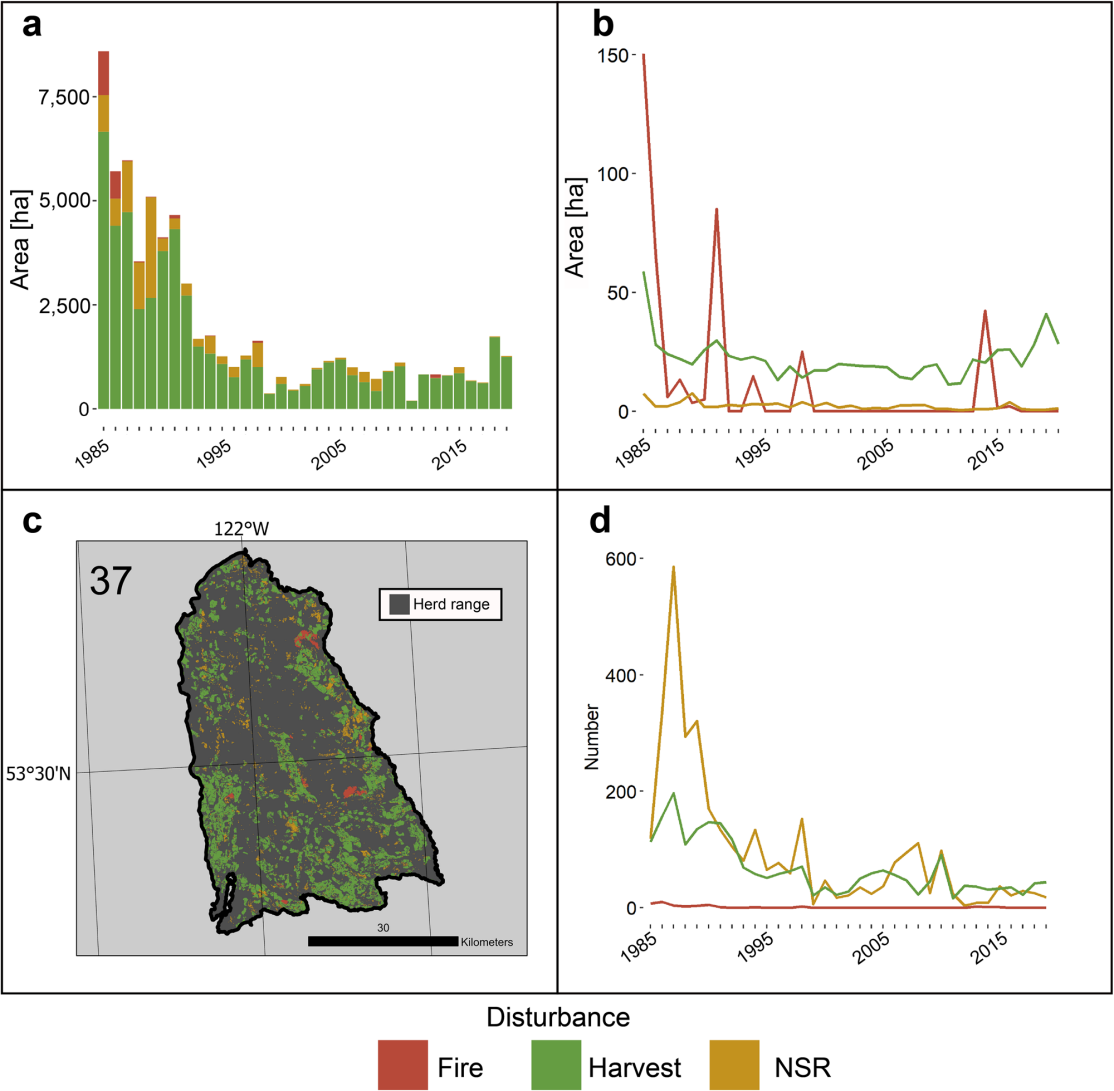
Annual disturbance dynamics for Narrow Lake herd from 1985 to 2019, (**a**) Area disturbed by each disturbance agent. (**b**) Average area of disturbance events. (**c**) Map of disturbance events across herd range, number indicating herd number. (**d**) Number of disturbance events per year.

**Figure 6:**
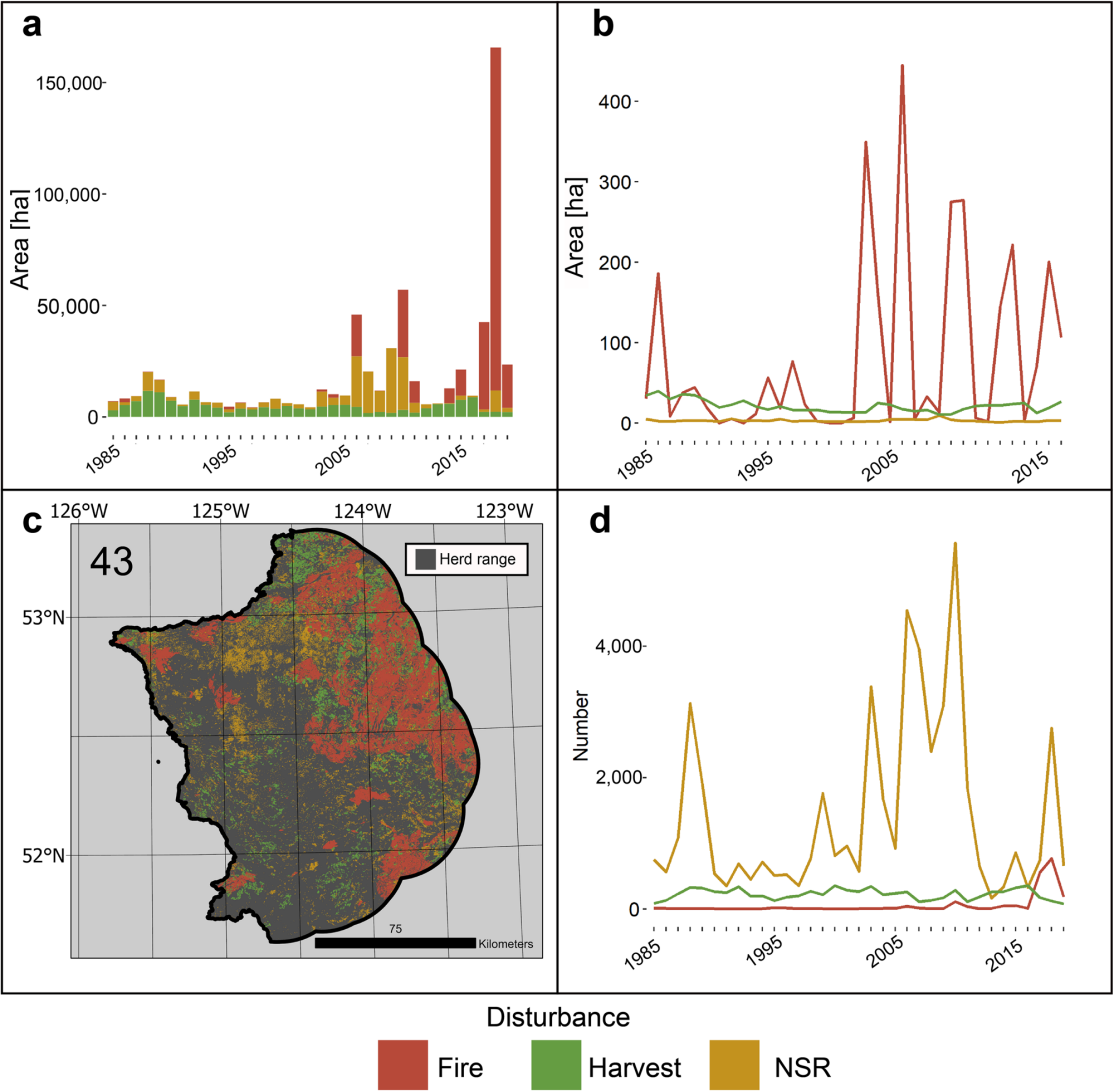
Annual disturbance dynamics for Itcha-Ilgachuz herd from 1985 to 2019, (**a**) Area disturbed by each disturbance agent. (**b**) Average area of disturbance events. (**c**) Map of disturbance events across herd range, number indicating herd number. (**d**) Number of disturbance events annually.

**Figure 7:**
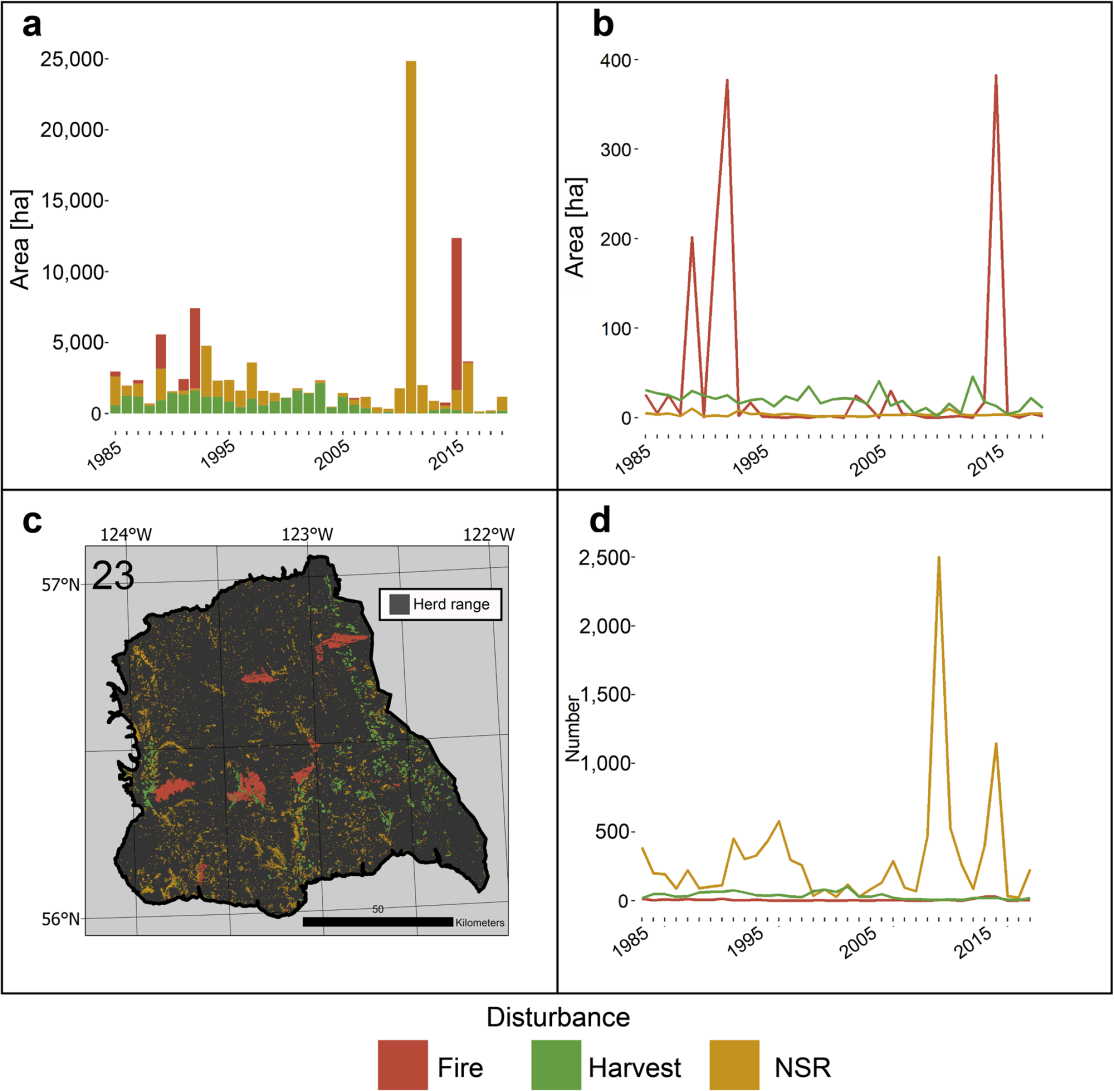
Annual disturbance dynamics for Graham herd from 1985 to 2019: (**a**) Area disturbed in thousands of hectares by each disturbance agent. (**b**) Average area of disturbance events in each year. (**c**) Map of disturbances across herd range, number indicating herd number. (**d**) Number of disturbance events of each type for each year.

Three herds with large, recent, population declines are described: the Narrow Lake (82.9% decrease from 2014 to 2020; Fig. 5), Itcha-Ilgachuz, (69.9% decrease from 2014 to 2020; Fig. 6), and Graham herds (69.1% decrease from 2009 to 2021; Fig. 7). Each of these herds highlights a different pattern of disturbance, with the Narrow Lake herd affected by large levels of harvesting (Fig. 5), the Itcha-Ilgachuz herd affected by large levels of fire as well as large amounts of harvesting (Fig. 6), and the Graham herd affected by relatively less disturbance (Fig. 7).

The Narrow Lake herd was affected by large amounts of disturbance, with 29% of its forested area disturbed within the study period. Harvesting was by far the main disturbance agent, with 24.1% of forested area harvested over the study period, equating to an average of 0.69% of forested area annually harvested (Fig. 5). This level of harvesting was over double the average (0.3%) for other Southern Mountain Southern group herds. The herd experienced very little fire, at 0.03% of forested area disturbed annually. This is consistent with other herds in the Southern group of Southern Mountain caribou, at an average of 0.05%. Non-stand replacing disturbance affected an average of 0.13% of forested area annually, commensurate with the 0.15% experienced for other herds of this group and ecotype.

The Itcha-Ilgachuz herd was affected by very high levels of disturbance, with 40.6% of forested area undergoing some type of disturbance over the study period. Fire has been a dominant disturbance in recent years with NSR being a major disturbance agent in the 2000’s (Figure 6). Fire has disturbed 18.6% of forested area over the study period, equating to an average of 0.53% of forested area annually, more than the 0.33% of forested area annually typically disturbed by fire for Southern Mountain Northern group caribou overall. Number of fire events remained consistent throughout the study period, with area disturbed by fire being modulated by increases in average fire size. 10.5% of the herd’s forested area was harvested over the study period, equating to an annual average of 0.3%, slightly higher than the 0.25% rate for Southern Mountain Northern group caribou overall.

Despite a large decline in population of 69.1% from 2009 to 2021, the Graham herd has experienced relatively low polygonal disturbance levels, with only 13% of forested area being disturbed over the study period. Harvesting was a major disturbance agent until the mid 2000’s, when it decreased, and non-stand replacing disturbance events increased in frequency (Fig. 7). This is consistent with the timing of the occurrence of the mountain pine beetle outbreak in BC^56^. On average, 0.09% of forested area in the herd range was affected by harvesting annually, less than half the 0.25% average for Southern Mountain Northern group caribou overall.

## Discussion

Despite a high public profile and a number of management actions taken with the focus of minimizing disturbance impacts, caribou populations are still declining across BC^18,70^. Urgent action and effective policy implementation are needed to ensure caribou populations do not continue to decline, guided by a comprehensive understanding of the disturbance dynamics related to this decline. In this study we quantified the polygonal disturbance regimes affecting caribou in BC and examined their characteristics over a long time-horizon. The results of our study indicate that caribou in BC face high, and in many cases increasing, levels of disturbance, and that to date, national-level recovery strategies and policies have been largely ineffective at preserving habitat, including during the lag period between SARA implementation in 2004 and recovery strategy publication and implementation in 2012/2014.

Southern Mountain ecotype caribou experienced much higher rates of disturbance than either Boreal or Northern Mountain ecotypes. These results align with the fact that multiple Southern Mountain Caribou herds have been extirpated or in steep decline over the recent decades, and underline the importance of reducing disturbance levels if conservation of these herds is to be successful^70^. Many Southern Mountain ecotype Southern group herds experienced relatively low levels of natural disturbance and elevated levels of harvest. In contrast, much lower levels of disturbance, especially anthropogenic, were found for Northern Mountain and Boreal herds. This study did not consider the impacts of linear disturbances, and thus true anthropogenic disturbance levels are likely higher than reported.

Northern Mountain, Boreal, and Southern Mountain Southern group caribou all experienced a net decreasing trend in harvesting levels over the study period. Given that human caused disturbances have been shown to be more detrimental to caribou than naturally occurring ones^12,21^, this may be heartening for land managers. However, in the most recent epoch (2010–2019), all three groups of Southern Mountain caribou experienced a moderate to large uptick in harvesting. This is likely due to an increase in annual allowable cut in these years, as a part of salvage logging efforts in response to beetle attack^51,55^. Salvage harvesting generates additional negative impacts for caribou on top of pre-existing disturbance, through reductions in lichen availability, and increases in linear disturbance, and thus poses a cumulative threat to caribou habitat on top of the pre-existing beetle attack^15,78^. The consequences of this increase in harvesting for declining populations need to be assessed, especially as this is the caribou ecotype in BC already facing the highest levels of anthropogenic disturbance.

In addition to quantifying levels of fire and harvesting, our study also quantified levels of NSR, an important variable when assessing habitat change for woodland caribou in BC, due to the large outbreak of mountain pine beetle which has occurred in the province^54,79^. While direct negative impacts of beetle attack and NSR on caribou habitat seem to be limited, the full ramifications of this outbreak on woodland caribou in BC are not fully understood^80,81^. This mountain pine beetle outbreak altered fire regimes^57,82^, as well as increased salvage logging^78^, which can negatively affect caribou, and further study on these impacts is required. NSR may therefore be an important predictor of upcoming stand-replacing disturbance. Given the levels of NSR across the province, effective policy to preserve caribou habitat must focus on minimizing the impacts and occurrence of these follow-on stand replacing disturbances.

We also examined the impact of SARA implementation and recovery strategy publication on harvesting levels. SARA was fully implemented in 2004, and recovery strategies for Boreal and Southern Mountain caribou were published in 2012 and 2014, designating critical habitat. However, SARA and listing of critical habitat only provide direct protection on federal lands^70^.

Statistically significantly lower harvesting levels were observed for Boreal and Northern Mountain caribou ecotypes following the implementation of SARA in 2004, however these are likely tied to external market forces rather than conservation policy. This decline did not occur immediately, but rather a few years after the implementation of SARA (Fig. 4). These declines coincide with a province-wide decrease in harvesting levels associated with the great recession^51,83^. Previous research has found that declines in harvesting in critical habitat for Boreal caribou were better explained by US timber markets than by the implementation of SARA, and this phenomenon is likely to apply to Northern Mountain caribou as well^84^.

We found no statistically significant decrease in harvesting levels for any group of Southern Mountain caribou after the implementation of SARA in 2004. This is notable, because herd ranges for these ecotypes undergo by far the most harvesting of any ecotype in the province, suggesting that the lag between SARA implementation and the subsequent identification and then protection of critical habitat should be of concern^10^. These findings align with the results of other studies which indicate that economic forces still likely influence harvesting activity in caribou range far more than conservation objectives, and that effective implementation of policy will require more effective prioritization of habitat protection^47,84^.

We found no statistically significant decrease in harvesting levels after the implementation of either of the recovery strategies for Southern Mountain and Boreal ecotype caribou, and the management plan for Northern Mountain caribou, indicating these policies are not yet successful in their goal of preserving caribou habitat. Our results indicate that Southern Mountain caribou, the herds most affected by anthropogenic disturbance have seen increased harvesting in the past decade. It is likely that these policies have as of yet had little effect on limiting harvesting levels for any ecotype of caribou in BC.

Disturbance patch dynamics observed varied by disturbance agent, caribou herd and ecotype. Harvesting events were typically smaller and more numerous than fire events, matching previous findings on the subject, and indicating that they are more likely to cause habitat fragmentation^85^. Our results indicated that NSR was characterized by vastly more, but much smaller disturbance events than fire or harvesting. This follows an ecological understanding of NSR, wherein stands are often made up of trees of differing levels of vulnerability and different species, and thus forested areas experiencing NSR often end up as a mosaic of differing ages, species, and levels of disturbance^86,87^. It is possible that, due to the non-stand replacing nature, and mosaic of differing age classes and species created by this disturbance, habitat fragmentation impacts are less than the small event size and large number of disturbance events would imply.

Many studies have evaluated disturbance levels affecting different caribou ecotypes and herds in Canada and BC^10,12,70,84,88^. Most of these have used polygonal data from forestry records and fire databases. While sufficient to characterize overall disturbance levels, the underlying data are derived from different sources with different criteria and levels of detail^28,30,58,89^. Long time series of calibrated remotely sensed data, such as from the Landsat satellite series, enable the wall-to-wall detection and characterization of disturbances, using the same methodology, criteria, and level of detail. By using a consistent disturbance identification methodology, the disturbance-derived metrics presented here allow for direct comparisons of disturbance dynamics across all herds in BC, as the disturbance information is not subject to potential variations due to different data sources, reporting requirements, and identification criteria^58,89^. Landsat-derived disturbance data also allows for a deep evaluation of the patch dynamics affecting caribou, with the relatively fine resolution of disturbance detection allowing for identification of small disturbances which may be missed, as well as remnant undisturbed areas within larger disturbances^30^.

Landsat-derived disturbance maps such as the one used in this study provide transparent and openly available, methodologically consistent, wall-to-wall depictions of forest change. As with any disturbance product, these maps are not without some level of error and associated uncertainty. The overall accuracy of the disturbance product utilized was 90%. Fire and harvesting were detected more reliably (> 95% detection) than NSR disturbances (83%)^58^. However, accuracy can vary depending on location, size, and harvesting practice. For example, clear-cut harvesting is more accurately detected than other types of harvesting such as shelterwood harvesting systems^90^. As a result, although there is some uncertainty in the disturbance product utilized, it has been quantified in peer-reviewed publications and provides a consistent and validated basis for landscape-level disturbance analysis.

Critically the disturbance information used in this paper is also updated annually^91^, providing not only consistent assessment of disturbances not only at a spatial scale but also over time. As a result, changes in the rate of disturbances can be monitored annually, automatically without the need to wait for new aerial imagery, human photointerpretation, or the transfer of records from forestry companies to the Province.

Linear disturbances, such as roads and seismic lines, are detrimental to woodland caribou, allow for more efficient predator movement, and are strongly associated with caribou population declines^10,92^. The 30-m spatial resolution of the Landsat data used limits the consistent detection of linear disturbances^93^. As a result, our analysis does not comprehensively assess all major disturbances affecting caribou in the province and focuses solely on polygonal disturbance. Therefore, total levels of anthropogenic disturbance affecting caribou are higher than reported in this study. The detection of linear disturbances requires the use of higher spatial resolution data. High-resolution satellite data could be used to generate a snapshot of current linear disturbance levels; however imagery is not available throughout the complete study period. Data from airborne laser scanning (ALS) has also been shown to be effective at identifying linear disturbance, and with the BC government’s announcement of a plan to collect provincial wall-to-wall ALS data, this may be feasible within the next few years^94,95^, however, this will also only provide a snapshot of current linear disturbance levels, and long time-series analysis using this data will not be possible.

Nagy-Reis et al.^42^ previously used a satellite-derived forest change data product to estimate stand-replacing disturbance in caribou habitat in Alberta and BC, at subpopulation level from 2000–2018. Our results quantifying annual stand-replacing disturbance levels were generally commensurate for Northern Mountain and Boreal ecotype caribou, as well as Southern Mountain Central and Northern group caribou. For Southern Mountain Southern group caribou, our results differed from those of Nagy-Reis et al.^42^. Our results indicated that Southern group caribou were affected by larger levels of stand-replacing disturbance when compared to results outlined in Nagy-Reis et al^42^. For Southern group caribou, 0.36% of forested area annually was affected by stand-replacing disturbance, compared to 0.24% of forested area found in Nagy-Reis et al^42^. This is mostly attributed to differences in levels of harvesting, with our study finding that 0.27% of forested area annually was harvested, compared to 0.18% in Nagy-Reis et al.^42^. These differences could be attributed to different factors, including the study periods (2000–2018 vs. 1985–2019), and differences in detection rates in the underlying disturbance products used. For instance, our study utilized a forest disturbance product designed in the context of Canadian forests, with integrated disturbance attribution, while Nagy-Reis et al.^42^ cross-referenced a global forest cover dataset^96^ with a secondary disturbance attribution product^97^

The three herds used as examples in our study (i.e., Narrow Lake, Itcha-Ilgachuz, Graham; Figs. 5, 6, and 7) have experienced very large population declines, but are affected by different disturbance regimes. The Itcha-Ilgachuz herd is affected by high, but standard for its ecotype levels of harvesting. However, it has seen massive levels of fire, much of it in the last decade (Fig. 7), and a consequent 69.9% decline in population from 2014 to 2020. This underlines that while fire is generally less detrimental to caribou than anthropogenic disturbance^10,12^, when large fires occur in herd ranges which already experience high levels of anthropogenic disturbance, significant declines can result. These large fires, linked to the history of fire suppression in the province, are likely to increase due to climate change. Management for herds at risk of these large fires should therefore balance fire mitigation strategies such as prescribed fires and fuel reductions with maintaining a mature forest structure^52^.

In contrast, the Narrow-Lake herd was affected by very little fire. However, very large areas of the herd’s range were harvested in the study period. The decline of this herd is likely simply related to the large level of harvesting present^10^. Conservation for herds affected by this level of harvesting requires effective policy to limit harvest and preserve Critical Habitat; as the results of our research, as well as those of Nagy-Reis et al.^42^, indicate that current policies have not been successful thus far.

The Graham herd has similarly large population declines, but has been affected by much less polygonal disturbance. It is therefore likely, that factors other than areal disturbances are the main drivers of this decline, such as linear disturbance or other industrial activities^46^. This herd is an example of a herd for which managers should likely focus less on minimizing gross levels of harvesting, or fire prevention, and rather identify and mitigate other causes of decline.

An accurate and detailed understanding of the disturbance regimes affecting caribou herds is critical for effective management. For example, a herd experiencing most of it’s habitat loss from harvesting is likely to be more negatively impacted, and may require different management strategies, than a herd primarily impacted by fire^12^. For herds with harvesting as a major disturbance driver, management actions may involve reducing allowable cut^84^. The impacts of a large fire year or insect attack within a herd range can be mitigated to some degree by decreasing allowable cut in forest not affected by those disturbances. Preserving habitat for herds with disturbance regimes dominated by fire may ultimately be more challenging, requiring balancing the ecological need for fire within historically fire-prone ecosystems affected by years of fire suppression with the need to maintain old-growth forest. Herds with disturbance regimes dominated by fire rather than harvest may require management tailored to prevent large, stand-replacing fires and instead focused on restoring historical frequent and low-severity fire regimes, through activities like fuel management and prescribed burning^52^.

Information on the rate of change also informs management. A herd that has experienced slightly less disturbance, but for which disturbance rates are increasing is likely to be a higher priority for urgent action than a herd that is currently experiencing slightly larger levels of disturbance, but for which annual area disturbed is decreasing.

## Conclusion

Our results have significant implications for caribou habitat management and science. The future of Southern Mountain caribou is a worrying one, with our results reinforcing that many herds have been affected by very high levels of both anthropogenic and natural disturbance, and that thus far, the impact of federal policies such as SARA on critical caribou habitat disturbance are not identifiable in the data for our study period. In caribou ranges where harvesting is a main disturbance driver, the lag between SARA implementation and critical habitat identification and subsequent protection is of concern. For many Southern Mountain herds, harvesting levels have increased in the past decade. Boreal and Northern Mountain caribou have been affected by relatively less polygonal disturbance; however, many herds continue to decline. To ensure a long-term future for caribou in BC, the root causes of their decline must be addressed, particularly habitat disturbance. Detailed disturbance information is critical to inform effective policy implementation. Herds with high rates of habitat alteration must have management actions tailored to their specific disturbance dynamics^16,17^. Without the implementation of effective policy that manages to lower disturbance levels, especially anthropogenic disturbance such as forest harvesting, it is likely that caribou populations will continue to decline.

## Supplementary Information

Supplementary Tables.

## Data Availability

Herd level results are included in this published article (and its supplementary information files). All other datasets generated or analysed during this study are available from the corresponding author on reasonable request.
