# Author Correction: Quantifying forest disturbance regimes within caribou (*Rangifer tarandus*) range in British Columbia

**DOI:** 10.1038/s41598-024-80617-6

**Published:** 2025-01-08

**Authors:** James C. Maltman, Nicholas C. Coops, Gregory J. M. Rickbeil, Txomin Hermosilla, A. Cole Burton

**Affiliations:** 1 Department of Forest Resources Management, Faculty of Forestry, University of British Columbia, Vancouver, BC Canada; 2 Ecofish Research, Suite 303-2012 Washington Street, Rossland, BC V0G 1Y0 Canada; 3 Canadian Forest Service (Pacific Forestry Centre), Natural Resources Canada, 506 West Burnside Road, Victoria, BC V8Z 1M5 Canada

Correction to: *Scientific Reports* 10.1038/s41598-024-56943-0, published online 19 March 2024

The original version of this Article contains errors, where herds were incorrectly assigned to the wrong ecotype for ecotype level analysis. The Tweedsmuir, Itcha-Ilgachuz, Rainbows, Charlotte Alplands, and Chase herds were incorrectly assigned to the Southern Mountain Central group when they instead should have been assigned to the Southern Mountain Northern group. This means that ecotype-level data on disturbance regimes for the Southern Mountain Northern and Central groups is inaccurate, mainly affecting fire and non-stand replacing disturbance rates.

Therefore, in the results section, the paragraph reading as follows:

Over the study period (1985–2019), a total of 16.4% of forested area was disturbed over all caribou herd ranges. For Southern Mountain caribou, 22.8% (3,641,500 ha) of forested area was disturbed, 9.0% (817,999 ha) of forested area was disturbed for Northern Mountain caribou, and 6.6% (238,363 ha) of forested area was disturbed for Boreal caribou. Southern Mountain Central group caribou were affected by the most disturbance of any Southern Mountain group, at 31.15% of forested area (55,742 ha). Boreal, Northern Mountain, and Southern Mountain Central group caribou had disturbance regimes primarily driven by fire, while Southern Mountain Northern, and Southern Mountain Southern group caribou had disturbance regimes dominated by harvesting (Table 1).

Now reads:

Over the study period (1985–2019), a total of 16.4% of forested area was disturbed over all caribou herd ranges. For Southern Mountain caribou, 22.8% (3,641,500 ha) of forested area was disturbed, 9.0% (817,999 ha) of forested area was disturbed for Northern Mountain caribou, and 6.6% (238,363 ha) of forested area was disturbed for Boreal caribou. Southern Mountain Northern group caribou were affected by the most disturbance of any Southern Mountain group, at 29.48% of forested area (383,739 ha). Boreal, Northern Mountain, and Southern Mountain Northern group caribou had disturbance regimes primarily driven by fire, while Southern Mountain Central, and Southern Mountain Southern group caribou had disturbance regimes dominated by harvesting (Table 1).

Also the paragraph reading as follows:

Disturbance agents varied by group within the Southern Mountain ecotype. All three groups (Southern, Central, and Northern) had high levels of harvesting, between 0.25 and 0.3% of area annually. Moderate variation between groups was present in levels of NSR, at 0.15% of area annually for the Southern group, 0.21% for the Central group, and 0.25% for the Northern group. Annual area disturbed by fire varied widely, with 0.38% of area being annually disturbed by fire for the Central group, but only 0.05% of area annually disturbed by fire for both Northern and Southern groups. An increase in harvesting levels in the last decade was found for all three groups, with the Northern group seeing the largest at 9.92% (Table 1).

Now reads:

Disturbance agents varied by group within the Southern Mountain ecotype. All three groups (Southern, Central, and Northern) had high levels of harvesting, between 0.25% and 0.3% of area annually. Moderate variation between groups was present in levels of NSR, at 0.15% of area annually for the Southern group, 0.14% for the Central group, and 0.26% for the Northern group. Annual area disturbed by fire varied widely, with 0.33% of area being annually disturbed by fire for the Northern group, but only 0.05% of area annually disturbed by fire for the Southern group. An increase in harvesting levels in the last decade was found for all three groups, with the Southern group seeing the largest at 6.67% (Table 1).

And the paragraph reading as follows:

The Itcha-Ilgachuz herd was affected by very high levels of disturbance, with 40.6% of forested area undergoing some type of disturbance over the study period. Fire has been a dominant disturbance in recent years with NSR being a major disturbance agent in the 2000’s (Fig. 6). Fire has disturbed 18.6% of forested area over the study period, equating to an average of 0.53% of forested area annually, an order of magnitude more than the 0.05% of forested area annually typically disturbed by fire for Southern Mountain Northern group caribou overall. Number of fire events remained consistent throughout the study period, with area disturbed by fire being modulated by increases in average fire size. 10.5% of the herd’s forested area was harvested over the study period, equating to an annual average of 0.3%, slightly higher than the 0.25% rate for Southern Mountain Northern group caribou overall.

Now reads:

The Itcha-Ilgachuz herd was affected by very high levels of disturbance, with 40.6% of forested area undergoing some type of disturbance over the study period. Fire has been a dominant disturbance in recent years with NSR being a major disturbance agent in the 2000’s (Figure 6). Fire has disturbed 18.6% of forested area over the study period, equating to an average of 0.53% of forested area annually, more than the 0.33% of forested area annually typically disturbed by fire for Southern Mountain Northern group caribou overall. Number of fire events remained consistent throughout the study period, with area disturbed by fire being modulated by increases in average fire size. 10.5% of the herd’s forested area was harvested over the study period, equating to an annual average of 0.3%, slightly higher than the 0.25% rate for Southern Mountain Northern group caribou overall.

In the Discussion, the paragraph reading as follows:

Nagy-Reis et al.^42^ previously used a satellite-derived forest change data product to estimate stand-replacing disturbance in caribou habitat in Alberta and BC, at subpopulation level from 2000 to 2018. Our results quantifying annual stand-replacing disturbance levels were generally commensurate for Northern Mountain and Boreal ecotype caribou, as well as Southern Mountain central group caribou. For Southern Mountain Northern and Southern group caribou, our results differed from those of Nagy-Reis et al.^42^. Our results indicated that Southern group caribou were affected by larger levels of stand-replacing disturbance, while Northern group caribou were affected by lower levels of stand-replacing disturbance when compared to results outlined in Nagy-Reis et al.^42^. For Southern group caribou, 0.36% of forested area annually was affected by stand-replacing disturbance, compared to 0.24% of forested area found in Nagy-Reis et al.^42^. This is mostly attributed to differences in levels of harvesting, with our study finding that 0.27% of forested area annually was harvested, compared to 0.18% in Nagy-Reis et al.^42^. For Northern group caribou, disturbance levels were almost half those found in Nagy-Reis et al.^42^, with our study finding 0.3% of forested area to be annually affected by disturbance, compared to 0.65% found in the other study. These differences could be attributed to different factors, including the study periods (2000–2018 vs. 1985–2019), and differences in detection rates in the underlying disturbance products used. For instance, our study utilized a forest disturbance product designed in the context of Canadian forests, with integrated disturbance attribution, while Nagy-Reis et al.^42^ cross-referenced a global forest cover dataset^96^ with a secondary disturbance attribution product^97^.

Now reads:

Nagy-Reis et al.^42^ previously used a satellite-derived forest change data product to estimate stand-replacing disturbance in caribou habitat in Alberta and BC, at subpopulation level from 2000–2018. Our results quantifying annual stand-replacing disturbance levels were generally commensurate for Northern Mountain and Boreal ecotype caribou, as well as Southern Mountain Central and Northern group caribou. For Southern Mountain Southern group caribou, our results differed from those of Nagy-Reis et al.^42^. Our results indicated that Southern group caribou were affected by larger levels of stand-replacing disturbance when compared to results outlined in Nagy-Reis et al^42^. For Southern group caribou, 0.36% of forested area annually was affected by stand-replacing disturbance, compared to 0.24% of forested area found in Nagy-Reis et al^42^. This is mostly attributed to differences in levels of harvesting, with our study finding that 0.27% of forested area annually was harvested, compared to 0.18% in Nagy-Reis et al.^42^. These differences could be attributed to different factors, including the study periods (2000–2018 vs. 1985–2019), and differences in detection rates in the underlying disturbance products used. For instance, our study utilized a forest disturbance product designed in the context of Canadian forests, with integrated disturbance attribution, while Nagy-Reis et al.^42^ cross-referenced a global forest cover dataset^96^ with a secondary disturbance attribution product^97^.

Correct data on disturbance levels for the two groups is wrongly represented in Table 1 and Figure 4.

The original Table 1 and its caption appear below:

**Table 1:** Mean annual area disturbed by disturbance agent, mean annual number of disturbance events, mean size of disturbance events, and Theil-Sen Test results for the three caribou ecotypes in British Columbia.

Ecotype	Mean annual area [ha/year]	Percent annual area [%/year]	Disturbance cycle [years]	Rate of change [ha/year]			Mean annual number of events	Rate of change 1985–2019 [count/year]	Mean event size [ha]	Rate of change 1985–2019 [ha/year]
				2010–2019	2000–2009	1985–2019				
Fire										
Boreal	4400.7	0.12%	819			36.99 (0.82%)	51	1.5 (2.93%)	205.6	3.6 (1.73%)
Northern Mountain	12,341.6	0.14%	733			133.92 (1.06%)	102		582.0	
Southern Mountain Northern	1514.3	0.05%	1973				184	3.3 1.81%)	329.2	
Southern Mountain Central	24,016.1	0.38%	264			191.41 (0.77%)	16		133.3	
Southern Mountain Southern	3474.3	0.05%	1914				77		255.2	
Harvesting										
Boreal	1327.0	0.04%	2715			− 53.98 (− 4.1%)	73	− 1.8 (− 2.39%)	58.1	− 1.4 (− 2.42%)
Northern Mountain	1135.7	0.01%	7962		− 222.68 (− 19.74%)	− 43.47 (− 3.85%)	87		79.8	− 1.4 (− 1.82%)
Southern Mountain Northern	7557.8	0.25%	395	758.43 (9.92%)	− 582.3 (− 7.62%)		938		188.8	
Southern Mountain Central	16,179.1	0.26%	391	583.73 (3.57%)			425		94.3	
Southern Mountain Southern	19,658.1	0.30%	338	1286.57 (6.67%)	− 897.34 (− 4.65%)	− 354.45 (− 1.84%)	1353	− 16.5 (− 1.22%)	251.5	
Non-stand replacing										
Boreal	1082.7	0.03%	3328				442		10.9	
Northern	9894.0	0.11%	914				3069		46.0	
Southern Mountain Northern	6341.1	0.21%	471	− 884.27 (− 14.06%)	141.88 (2.26%)		3926.5		29.9	
Southern Mountain Central	15,547.5	0.25%	407				1379.2		14.5	
Southern Mountain Southern	9754.5	0.15%	682			− 326.1 (− 3.51%)	2760.3	− 109.1 (− 3.95%)	44.4	

The original Figure 4 and its caption appear below:

**Fig. 4:**
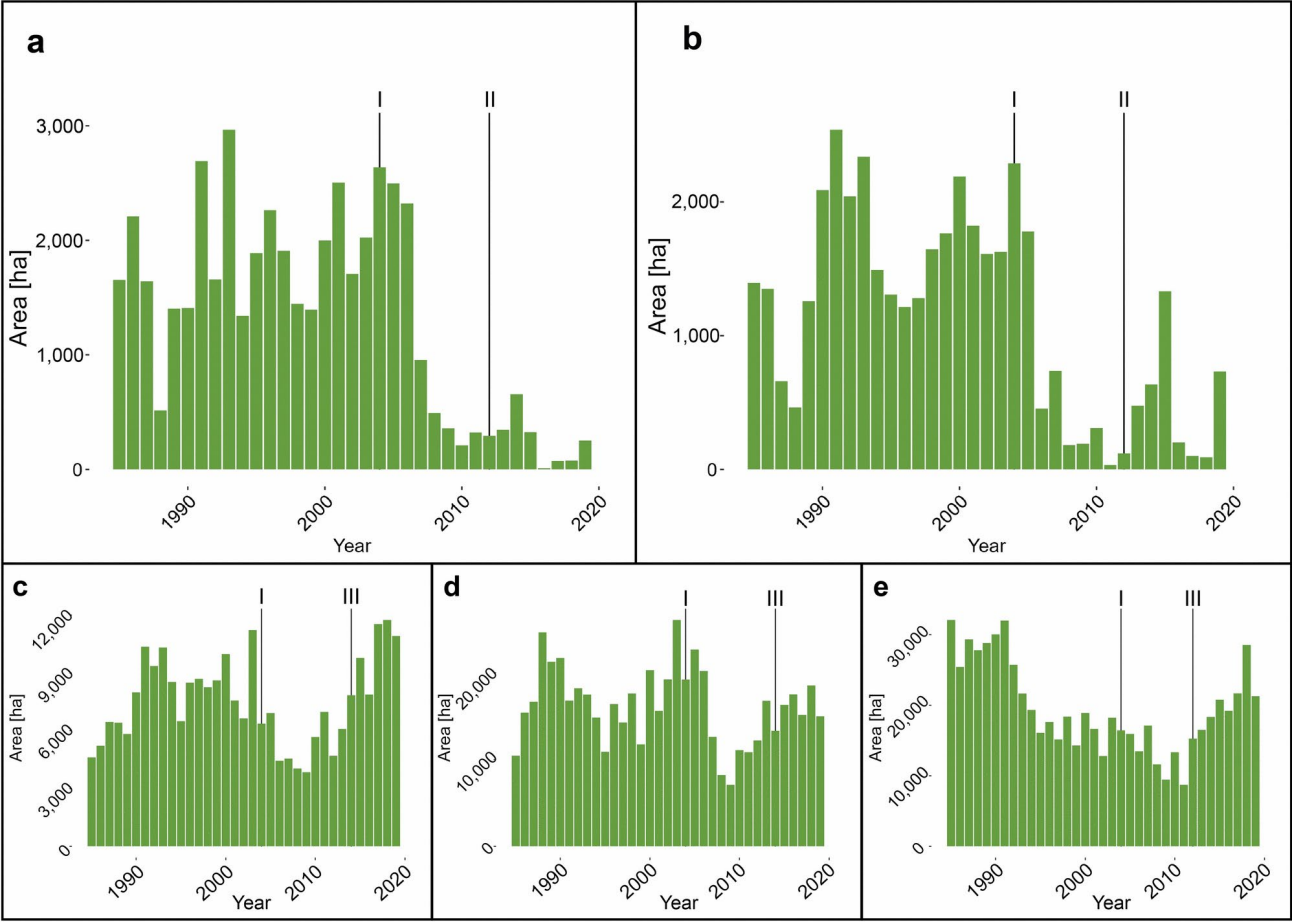
Annual area harvested for (**a**) Boreal, (**b**) Northern Mountain, (**c**) Southern Mountain Northern, (**d**) Southern Mountain Central, and (**e**) Southern Mountain Southern caribou. Lines representing: I: Full implementation of the Species at Risk Act in 2004, II: 2012 publishing of management plan for Northern Mountain caribou and recovery strategy for Boreal caribou, III: 2014 publishing of recovery strategy for Southern Mountain caribou.

The original Article has been corrected.

