# Supplementary material for: Quantifying forest disturbance regimes within caribou (Rangifer tarandus) range in British Columbia
Source: Sci Rep. 2024 Mar 19;14:6520. doi: 10.1038/s41598-024-56943-0 (PMC10948814; doi:10.1038/s41598-024-56943-0)
Supplement: Supplementary file 1 — Supplementary Tables. [file 41598_2024_56943_MOESM1_ESM.docx]

**Supplementary Table 1:**

**Data on fire regimes affecting BC caribou at herd level. Rate of change only presented for statistically significant (P<0.05) trends. Percent rate of change defined as rate of change in ha divided by average annual area disturbed.**

| Ecotype | Mean Annual Area  [ha/year] | Percent Annual Area  [%/year] | Fire  Cycle [years] | Rate of change [ha/year] | | | Mean Annual Number of Events | Mean Event Size [ha] |
| --- | --- | --- | --- | --- | --- | --- | --- | --- |
|  |  |  |  | **2010-2019** | **2000-2009** | **1985-2019** |  |  |
| Atlin | 83.0 | 0.02% | 4740 |  |  |  | 4 | 212.9 |
| Barkerville | 117.8 | 0.04% | 2427 |  |  | -2.15 (-1.82%) | 4 | 423.8 |
| Burnt Pine | 33.3 | 0.02% | 4592 |  |  | 0.75 (2.26%) | 3 | 133.1 |
| Calendar | 1104.3 | 0.37% | 269 |  |  | 1.26 (0.11%) | 11 | 695.8 |
| Carcross | 2.4 | 0.00% | 73765 |  |  |  | <1 | 174.2 |
| Central Rockies | 48.2 | 0.04% | 2236 |  |  | -0.36 (-0.74%) | 2 | 172.4 |
| Central Selkirks | 460.6 | 0.09% | 1137 |  |  |  | 13 | 234.2 |
| Charlotte Alplands | 1385.0 | 0.51% | 197 |  |  |  | 13 | 598.6 |
| Chase | 1691.4 | 0.19% | 516 |  |  |  | 35 | 310.3 |
| Chinchaga | 1826.5 | 0.16% | 642 |  |  |  | 20 | 570.7 |
| Columbia North | 242.2 | 0.04% | 2333 |  |  |  | 6 | 170.5 |
| Columbia South | 168.8 | 0.06% | 1762 | 21.56 (12.77%) |  |  | 5 | 179.2 |
| Edziza | 558.4 | 0.48% | 207 |  |  |  | 3 | 1930.6 |
| Finlay | 257.2 | 0.04% | 2243 |  |  | -0.44 (-0.17%) | 3 | 570.8 |
| Frisby Boulder | 34.9 | 0.03% | 3488 |  |  |  | 1 | 174.5 |
| Frog | 495.1 | 0.18% | 566 |  | 2.36 (0.48%) |  | 9 | 403.5 |
| Gataga | 195.6 | 0.06% | 1607 |  |  |  | 2 | 716.3 |
| George Mountain | 0.1 | <0.01% | >10,000 |  |  |  | <1 | 26.0 |
| Graham | 595.2 | 0.08% | 1317 |  |  |  | 5 | 558.4 |
| Groundhog | 71.3 | 0.03% | 3213 |  |  |  | 3 | 506.3 |
| Hart Ranges | 45.0 | <0.01% | >10,000 |  |  |  | 2 | 226.2 |
| Horseranch | 5464.1 | 0.45% | 221 |  |  | 5.72 (0.1%) | 19 | 1548.5 |
| Itcha-Ilgachuz | 8485.4 | 0.53% | 188 |  |  | 9.97 (0.12%) | 56 | 987.0 |
| Kennedy Siding | 25.7 | 0.01% | 7246 |  |  |  | <1 | 574.4 |
| Level-Kawdy | 519.9 | 0.09% | 1098 |  |  | 0.15 (0.03%) | 4 | 1532.3 |
| Liard Plateau | 27.3 | 0.01% | >10,000 |  |  |  | 1 | 227.5 |
| Little Rancheria | 1059.1 | 0.25% | 405 |  |  |  | 6 | 838.0 |
| Maxhamish | 244.7 | 0.04% | 2309 |  |  | 0.22 (0.09%) | 4 | 675.4 |
| Moberly | 638.8 | 0.11% | 896 |  |  |  | 6 | 480.2 |
| Monashee | 124.4 | 0.04% | 2478 |  |  |  | 3 | 217.4 |
| Muskwa | 1030.0 | 0.08% | 1281 |  |  |  | 13 | 677.8 |
| Narraway | 1146.8 | 0.27% | 376 |  |  |  | 7 | 843.6 |
| Narrow Lake | 58.2 | 0.03% | 3941 |  |  |  | 1 | 384.1 |
| North Cariboo | 330.9 | 0.06% | 1617 |  |  |  | 4 | 442.0 |
| Pink Mountain | 373.4 | 0.06% | 1750 |  |  |  | 8 | 354.5 |
| Purcell Central | 425.1 | 0.11% | 920 | 23.51 (5.53%) |  | 0.8 (0.19%) | 6 | 290.3 |
| Purcells South | 560.0 | 0.17% | 606 | 34.47 (6.16%) |  |  | 6 | 299.9 |
| Quintette | 161.5 | 0.03% | 3190 |  |  | -2.37 (-1.47%) | 9 | 154.7 |
| Rabbit | 388.6 | 0.05% | 2120 |  |  | 1.75 (0.45%) | 6 | 561.8 |
| Rainbows | 1111.4 | 0.35% | 287 |  |  |  | 6 | 841.2 |
| Redrock-Prairie Creek | 16.2 | 0.01% | 8322 |  |  |  | <1 | 602.7 |
| Scott | 5.6 | <0.01% | >10,000 |  |  |  | <1 | 90.8 |
| Snake-Sahtaneh | 773.0 | 0.09% | 1151 |  |  | 3.74 (0.48%) | 9 | 434.5 |
| South Selkirks | 298.3 | 0.12% | 868 | 37.77 (12.66%) |  | 0.08 (0.03%) | 4 | 311.7 |
| Spatsizi | 740.2 | 0.09% | 1134 |  |  | 0.19 (0.03%) | 9 | 405.5 |
| Swan Lake | 855.6 | 0.24% | 414 |  |  |  | 7 | 555.1 |
| Takla | 35.9 | 0.02% | 5026 | 0.58 (1.63%) |  |  | 1 | 302.5 |
| Telkwa | 85.4 | 0.02% | 6013 |  |  |  | 1 | 1147.3 |
| Thutade | 290.4 | 0.07% | 1472 |  |  |  | 6 | 511.1 |
| Tsenaglode | 1.2 | <0.01% | >10,000 |  |  |  | <1 | 67.4 |
| Tweedsmuir | 9953.9 | 0.60% | 168 |  |  | 41.68 (0.42%) | 54 | 870.1 |
| Wells Gray North | 346.8 | 0.05% | 1941 |  |  |  | 8 | 326.5 |
| Wells Gray South | 141.8 | 0.02% | 5448 |  |  |  | 8 | 185.8 |
| Westside Fort Nelson | 452.2 | 0.07% | 1501 |  |  |  | 7 | 555.8 |
| Wolverine | 158.9 | 0.02% | 5894 |  |  |  | 3 | 204.2 |

**Supplementary Table 2:**

**Data on harvest regimes affecting BC caribou at herd level. Rate of change only presented for statistically significant (P<0.05) trends. Percent rate of change defined as rate of change in ha divided by average annual area disturbed.**

| Ecotype | Mean Annual Area  [ha/year] | Percent Annual Area  [%/year] | Harvest  Cycle [years] | Rate of change [ha/year] | | | Mean Annual Number of Events | Mean Event Size [ha] |
| --- | --- | --- | --- | --- | --- | --- | --- | --- |
|  |  |  |  | **2010-2019** | **2000-2009** | **1985-2019** |  |  |
| Atlin | 5.0 | <0.01% | >10,000 |  |  |  | 2 | 25.7 |
| Barkerville | 1632.3 | 0.57% | 175 |  |  |  | 90 | 205.3 |
| Burnt Pine | 541.0 | 0.35% | 283 |  |  | 27.2 (5.03%) | 26 | 214.6 |
| Calendar | 4.6 | <0.01% | >10,000 |  |  |  | 1 | 46.1 |
| Carcross | 1.6 | <0.01% | >10,000 |  |  |  | 1 | 29.8 |
| Central Rockies | 262.3 | 0.24% | 411 |  | -10.51 (-4.01%) | -10.39 (-3.96%) | 23 | 130.1 |
| Central Selkirks | 1004.3 | 0.19% | 521 | 69.11 (6.88%) |  | -12.92 (-1.29%) | 99 | 115.7 |
| Charlotte Alplands | 177.6 | 0.07% | 1537 |  | -25.36 (-14.28%) | -4.06 (-2.28%) | 12 | 105.4 |
| Chase | 1467.1 | 0.17% | 595 | 213.39 (14.54%) | -220.3 (-15.02%) | -65.38 (-4.46%) | 75 | 206.1 |
| Chinchaga | 275.6 | 0.02% | 4253 |  |  | -12.76 (-4.63%) | 19 | 138.0 |
| Columbia North | 1911.4 | 0.34% | 296 | 154.98 (8.11%) | -163.05 (-8.53%) | -44.36 (-2.32%) | 168 | 126.1 |
| Columbia South | 429.4 | 0.14% | 693 | -42.55 (-9.91%) |  |  | 41 | 113.8 |
| Edziza | 5.5 | <0.01% | >10,000 |  |  |  | 2 | 18.9 |
| Finlay | 515.1 | 0.09% | 1120 |  | -134.71 (-26.15%) | -29.02 (-5.63%) | 22 | 208.2 |
| Frisby Boulder | 409.8 | 0.34% | 297 |  |  |  | 35 | 127.8 |
| Frog | 1.9 | <0.01% | >10,000 |  |  |  | 1 | 20.2 |
| Gataga | 3.9 | <0.01% | <10,000 |  |  |  | 2 | 24.0 |
| George Mountain | 342.7 | 0.79% | 126 | 58.65  (17.12%) |  |  | 14 | 273.5 |
| Graham | 728.1 | 0.09% | 1076 |  | -188.27 (-25.86%) | -37.26 (-5.12%) | 35 | 214.5 |
| Groundhog | 1174.4 | 0.51% | 195 |  |  |  | 104 | 126.2 |
| Hart Ranges | 2364.0 | 0.24% | 409 | 439.74 (18.6%) | -301.68 (-12.76%) | -88.06 (-3.73%) | 96 | 258.9 |
| Horseranch | 23.3 | <0.01% | >10,000 |  |  |  | 8 | 45.1 |
| Itcha-Ilgachuz | 4741.0 | 0.30% | 336 |  |  |  | 225 | 235.3 |
| Kennedy Siding | 565.7 | 0.30% | 329 |  |  |  | 26 | 231.3 |
| Level-Kawdy | 6.5 | <0.01% | >10,000 |  | 0.11 (1.67%) |  | 2 | 40.9 |
| Liard Plateau | 0.7 | <0.01% | >10,000 |  |  |  | 1 | 11.6 |
| Little Rancheria | 14.1 | <0.01% | >10,000 |  |  |  | 3 | 46.9 |
| Maxhamish | 210.0 | 0.04% | 2690 |  |  |  | 11 | 190.0 |
| Moberly | 1601.0 | 0.28% | 358 |  | -160.29 (-10.01%) |  | 77 | 228.9 |
| Monashee | 1087.4 | 0.35% | 284 | 138.18 (12.71%) |  |  | 95 | 125.4 |
| Muskwa | 132.9 | 0.01% | 9930 |  |  |  | 8 | 169.5 |
| Narraway | 377.7 | 0.09% | 1142 |  |  |  | 24 | 171.1 |
| Narrow Lake | 1576.4 | 0.69% | 145 |  |  | -58.01 (-3.68%) | 67 | 245.4 |
| North Cariboo | 1230.2 | 0.23% | 435 |  | -62.88 (-5.11%) | -64.03 (-5.21%) | 52 | 212.3 |
| Pink Mountain | 28.9 | <0.01% | >10,000 |  |  |  | 5 | 66.1 |
| Purcell Central | 1088.7 | 0.28% | 359 | -205.32 (-18.86%) |  | 43.06 (3.96%) | 73 | 164.7 |
| Purcells South | 1125.9 | 0.33% | 301 |  |  |  | 98 | 134.8 |
| Quintette | 1309.4 | 0.25% | 393 |  |  |  | 67 | 225.9 |
| Rabbit | 20.7 | <0.01% | >10,000 |  |  |  | 6 | 33.3 |
| Rainbows | 393.8 | 0.12% | 810 | 64.98 (16.5%) | -82.35 (-20.91%) |  | 25 | 168.1 |
| Redrock-Prairie Creek | 98.6 | 0.07% | 1371 | 7.29 (7.4%) | -2.38 (-2.42%) |  | 6 | 186.3 |
| Scott | 1342.4 | 0.74% | 135 |  | -113.34 (-8.44%) |  | 61 | 250.6 |
| Snake-Sahtaneh | 108.6 | 0.01% | 8192 | -10.23 (-9.41%) |  |  | 10 | 97.6 |
| South Selkirks | 597.4 | 0.23% | 434 |  |  | 11.71 (1.96%) | 57 | 117.7 |
| Spatsizi | 34.2 | <0.01% | >10,000 |  |  |  | 8 | 55.4 |
| Swan Lake | 6.8 | <0.01% | >10,000 |  |  |  | 2 | 28.3 |
| Takla | 678.2 | 0.38% | 266 |  |  |  | 36 | 218.2 |
| Telkwa | 1995.1 | 0.39% | 257 |  |  |  | 155 | 145.8 |
| Thutade | 333.8 | 0.08% | 1281 |  | -92.9 (-27.84%) | -17.5 (-5.24%) | 15 | 264.0 |
| Tsenaglode | 1.0 | <0.01% | >10,000 |  |  |  | 1 | 14.9 |
| Tweedsmuir | 5164.8 | 0.31% | 324 |  |  |  | 391 | 162.1 |
| Wells Gray North | 1762.1 | 0.26% | 382 |  |  | -44.55 (-2.53%) | 103 | 187.3 |
| Wells Gray South | 1659.4 | 0.21% | 466 | 202.45 (12.2%) | -141.4 (-8.52%) |  | 139 | 133.7 |
| Westside Fort Nelson | 728.2 | 0.11% | 932 |  |  | -40.46 (-5.56%) | 32 | 227.5 |
| Wolverine | 2555.4 | 0.27% | 367 | 722.06 (28.26%) | -306.55 (-12%) |  | 121 | 240.6 |

**Supplementary Table 3:**

**Data on non-stand replacing disturbance regimes affecting BC caribou at herd level. Rate of change only presented for statistically significant (P<0.05) trends. Percent rate of change defined as rate of change in ha divided by average annual area disturbed.**

| Ecotype | Mean Annual Area  [ha/year] | Percent Annual Area  [%/year] | NSR  Cycle [years] | Rate of change [ha/year] | | | Mean Annual Number of Events | Mean Event Size [ha] |
| --- | --- | --- | --- | --- | --- | --- | --- | --- |
|  |  |  |  | **2010-2019** | **2000-2009** | **1985-2019** |  |  |
| Atlin | 332.5 | 0.08% | 1183 |  |  |  | 90.1 | 29.0 |
| Barkerville | 630.2 | 0.22% | 454 |  |  | -16.27 (-2.58%) | 182.9 | 33.6 |
| Burnt Pine | 190.5 | 0.12% | 802 | -61.73 (-32.4%) |  |  | 78.9 | 25.8 |
| Calendar | 41.6 | 0.01% | 7156 |  |  | 0.95 (2.3%) | 37.0 | 12.5 |
| Carcross | 137.9 | 0.08% | 1261 | -23.74 (-17.22%) |  |  | 42.4 | 26.5 |
| Central Rockies | 91.5 | 0.08% | 1178 |  |  | -2.43 (-2.65%) | 44.7 | 17.0 |
| Central Selkirks | 807.6 | 0.15% | 648 |  |  | -17.61 (-2.18%) | 262.1 | 26.6 |
| Charlotte Alplands | 164.1 | 0.06% | 1663 |  |  |  | 79.7 | 20.7 |
| Chase | 1438.3 | 0.16% | 607 |  |  |  | 412.2 | 33.1 |
| Chinchaga | 510.9 | 0.04% | 2294 |  |  | -12.78 (-2.5%) | 174.9 | 36.2 |
| Columbia North | 646.7 | 0.11% | 874 |  |  | -18.79 (-2.9%) | 244.9 | 25.1 |
| Columbia South | 382.3 | 0.13% | 778 |  |  | -4.1 (-1.07%) | 123.2 | 24.5 |
| Edziza | 45.5 | 0.04% | 2539 |  |  | 0.7 (1.53%) | 20.3 | 19.0 |
| Finlay | 1018.0 | 0.18% | 567 |  |  |  | 312.7 | 34.3 |
| Frisby Boulder | 160.5 | 0.13% | 759 |  |  | -2.29 (-1.42%) | 49.9 | 27.1 |
| Frog | 545.5 | 0.19% | 514 |  |  |  | 156.6 | 26.9 |
| Gataga | 536.4 | 0.17% | 586 |  |  |  | 188.8 | 29.4 |
| George Mountain | 42.3 | 0.10% | 1020 |  |  |  | 13.7 | 30.7 |
| Graham | 1596.9 | 0.20% | 491 |  | 43.51 (2.72%) |  | 304.6 | 39.1 |
| Groundhog | 247.1 | 0.11% | 927 | -17.48 (-7.07%) |  | -8.23 (-3.33%) | 93.4 | 25.1 |
| Hart Ranges | 1463.2 | 0.15% | 661 |  |  |  | 302.8 | 44.9 |
| Horseranch | 1300.5 | 0.11% | 930 |  |  |  | 379.9 | 36.1 |
| Itcha-Ilgachuz | 5281.3 | 0.33% | 302 |  | 1781.13 (33.73%) |  | 1437.4 | 33.7 |
| Kennedy Siding | 179.8 | 0.10% | 1036 |  |  |  | 61.0 | 27.1 |
| Level-Kawdy | 337.6 | 0.06% | 1691 |  |  | 9.19 (2.72%) | 153.9 | 20.1 |
| Liard Plateau | 228.7 | 0.05% | 1951 |  |  |  | 65.6 | 36.1 |
| Little Rancheria | 211.9 | 0.05% | 2023 |  |  |  | 82.1 | 28.2 |
| Maxhamish | 131.5 | 0.02% | 4297 |  |  | 1.47 (1.12%) | 50.7 | 21.5 |
| Moberly | 1214.6 | 0.21% | 471 |  |  |  | 260.2 | 34.1 |
| Monashee | 223.3 | 0.07% | 1380 |  |  | -8.64 (-3.87%) | 95.9 | 22.5 |
| Muskwa | 987.0 | 0.07% | 1337 |  |  |  | 296.1 | 32.4 |
| Narraway | 294.6 | 0.07% | 1464 |  |  |  | 81.2 | 34.0 |
| Narrow Lake | 294.6 | 0.13% | 778 |  |  | -13.99 (-4.75%) | 97.2 | 25.3 |
| North Cariboo | 972.5 | 0.18% | 550 | 75.31 (7.74%) |  | -25.77 (-2.65%) | 243.2 | 37.4 |
| Pink Mountain | 548.3 | 0.08% | 1192 |  | 63.16 (11.52%) |  | 142.2 | 33.2 |
| Purcell Central | 509.5 | 0.13% | 768 |  |  | -26.4 (-5.18%) | 172.5 | 27.8 |
| Purcells South | 210.5 | 0.06% | 1611 | -12.99 (-6.17%) |  | -8.21 (-3.9%) | 95.6 | 23.2 |
| Quintette | 366.2 | 0.07% | 1406 |  | 96.02 (26.22%) |  | 141.7 | 26.8 |
| Rabbit | 1316.3 | 0.16% | 626 |  |  |  | 421.4 | 35.3 |
| Rainbows | 634.2 | 0.20% | 503 |  |  |  | 159.8 | 29.8 |
| Redrock-Prairie Creek | 211.6 | 0.16% | 639 |  |  |  | 59.9 | 32.8 |
| Scott | 642.9 | 0.36% | 281 |  | 13.44 (2.09%) |  | 145.1 | 35.0 |
| Snake-Sahtaneh | 189.6 | 0.02% | 4694 |  |  |  | 105.1 | 18.4 |
| South Selkirks | 117.9 | 0.05% | 2197 |  | 9.76 (8.28%) |  | 48.9 | 21.3 |
| Spatsizi | 1329.3 | 0.16% | 632 |  |  |  | 396.1 | 34.0 |
| Swan Lake | 234.1 | 0.07% | 1513 |  |  |  | 84.9 | 29.8 |
| Takla | 372.6 | 0.21% | 485 |  |  |  | 100.1 | 32.7 |
| Telkwa | 329.1 | 0.06% | 1561 | -79.74 (-24.23%) | 31.57 (9.59%) |  | 169.8 | 21.4 |
| Thutade | 615.0 | 0.14% | 695 |  |  |  | 178.8 | 33.4 |
| Tsenaglode | 169.5 | 0.15% | 681 | 13.1 (7.73%) |  | 1.61 (0.95%) | 57.3 | 29.7 |
| Tweedsmuir | 6143.9 | 0.37% | 272 |  |  |  | 1269.6 | 34.2 |
| Wells Gray North | 1427.7 | 0.21% | 472 |  |  | -33.5 (-2.35%) | 287.4 | 45.6 |
| Wells Gray South | 1527.1 | 0.20% | 506 |  |  | -32.61 (-2.14%) | 401.9 | 38.9 |
| Westside Fort Nelson | 209.2 | 0.03% | 3245 |  |  | -5.33 (-2.55%) | 73.8 | 32.9 |
| Wolverine | 2827.9 | 0.30% | 331 |  |  |  | 544.5 | 33.5 |
